# Deformable Eutectic Alloy With Near‐Theoretical Yield Strength via Hierarchical Nanoscale Multiphases and Sessile Defects

**DOI:** 10.1002/advs.202518764

**Published:** 2026-01-05

**Authors:** Yusha Luo, Qianqian Wang, Bo Sun, Ruixin Sheng, Zhijun Guo, Gaopeng Zou, Zhe Jia, Yang Tong, Gang Sha, Peter K. Liaw, Baolong Shen

**Affiliations:** ^1^ School of Materials Science and Engineering Jiangsu Key Laboratory for Advanced Metallic Materials Southeast University Nanjing China; ^2^ Ministry of Education Key Laboratory of Structure and Thermal Protection for High‐Speed Aircraft Southeast University Nanjing China; ^3^ Advanced Studies in Precision Materials Yantai University Yantai China; ^4^ School of Materials Science and Engineering Herbert Gleiter Institute of Nanoscience Nanjing University of Science and Technology Nanjing China; ^5^ Department of Materials Science and Engineering The University of Tennessee Knoxville USA

**Keywords:** eutectic high‐entropy alloys, hierarchical nanoscale multiphases, nanoscale lamellae, near‐theoretical strength, sessile defects

## Abstract

Eutectic high‐entropy alloys (EHEAs), a typical bioinspired lamellar composite, have the potential to achieve high strength and good ductility simultaneously for structural applications through microstructure modification. However, an extreme modulus/hardness mismatch between constituent phases leads to premature fracture and severely limits the achievable yield strength by impeding plasticity at room temperature. Here, a CoCrFeNiTa_0.4_ EHEA designed via suction casting followed by precise thermal treatment, which exhibits sessile interface defects and hierarchical nano‐multiphase structures consisting of FCC‐Laves eutectic lamellae, L1_2_ and D0_22_ coprecipitates, attains a near‐theoretical yield strength of 2.6 GPa alongside sufficient plasticity of 13.6%. This breakthrough is attributed to multiple mechanisms, characterizing soft‐FCC nanolamellae strengthened by coherent L1_2_ precipitates, sessile planar faults, and misfit‐interface dislocations, while hard‐Laves nanolamellae are toughened by deformable D0_22_ precipitates. All of these factors lead to the reduced modulus/hardness mismatch between FCC and Laves lamellae. The results indicate that the long‐range modulus/hardness‐matching and short‐range heterostructure, via hierarchical multiple phases and defects, are pivotal for next‐generation dual‐ and multi‐phase alloys to achieve theoretical strength while retaining impressive plasticity.

## Graphical abstract

A CoCrFeNiTa_0.4_ eutectic high‐entropy alloy achieves a near‐theoretical yield strength of 2.6 GPa with 13.6% plasticity. This breakthrough stems from a hierarchical nanostructure (FCC‐Laves lamellae with L1_2_/D0_22_ precipitates), which alleviates the inter‐phase modulus/hardness mismatch through synergistic strengthening and toughening, guiding the design of next‐generation strong‐ductile alloys.

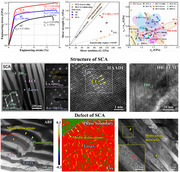

## 1 Introduction

Advanced structural materials with ultrahigh strength and sufficient plastic strain are highly desirable for sophisticated industrial components, such as aircraft landing gears, rocket cases, and high‐strength fasteners [1, 2]. Recent advancements in developing bioinspired composites, specifically eutectic high entropy alloys (EHEAs) [3, 4, 5], have emerged as promising candidates. EHEAs inherit lamellar structures, which are common structural elements in biological materials, such as cortical bone and nacre [6, 7, 8]. These structures consist of alternating assemblies of hard and soft phases, effectively providing resilience and strength to these materials. As reported, the strength‐optimization mechanism of cortical bone, achieved through the nanoscale platelet organization that reaches theoretical strength despite inherent defects [9], has been replicated in AlCoCrFeNi_2.1_ EHEA via laser power bed fusion [5]. This advanced manufacturing technique involves large‐temperature gradients and rapid cooling rates, which enable the microstructural refinement at the nanoscale that improves the yield strength to 1.6 GPa [5] by interphase boundary strengthening. Therefore, replicating the multiscale structures and load‐bearing functionality of biological materials enables biomimetic manufacturing to serve as a frontier approach for developing next‐generation high‐performance alloys.

However, a fundamental challenge to bioinspired composites is the extreme modulus mismatch and hardness disparity between the soft and hard components [10], which, along with the complex and costly powder metallurgy [5], collectively hinder the attainment of their theoretical strength and broader engineering applications. As proposed by Frenkel [11], the theoretical shear‐strength limit in single‐phase alloys is around *G*/10 (*G* is the shear modulus). For eutectic composites, the theoretical shear strength could be estimated by the rule of mixture (ROM) [12], $\sigma_{y}^{upper}=\sum_{i}V_{i}G_{y,i}\;$, where *V_i_* and *G*_*y*,*i*_ are the volume fraction and shear modulus of phase, *i*, respectively. The yield strength of widely studied EHEAs, comprising a dual‐phase microstructure of a soft phase (FCC phase: 200 HV [13, 14]) and harder intermetallic phases (B2 phase: 750 HV [15]; Laves phase: 744–1,700 HV [16]), rarely exceeds 1.8 GPa (∼*G*/100 far from theoretical strength, *G* is the shear modulus) [5, 17]. Particularly in FCC‐Laves EHEAs, severe modulus mismatch often induces premature cracks prior to yielding [17], resulting in embrittlement. Although gradient, bimodal, and core–shell heterogeneous nanostructures [18, 19, 20] demonstrating potential for hardness/modulus matching, the creation of these heterogeneities require the incorporation of some coarse‐scale microstructures that sacrifice the strength, and sophisticated processing requirements.

In this paper, we overcome these critical challenges and present the development of a new eutectic alloy with hierarchical multiple phases and sessile defects, which approaches theoretical strength with impressive plasticity, via suction casting followed by precise thermal treatment. Our design philosophy embodies a nuanced application of heterostructure principles, aiming for the mitigation rather than the complete elimination of detrimental heterogeneity. This involves two key steps: (i) to create nanometer‐thick lamellae avoiding coarse‐scale microstructures for a high‐strengthening purpose through suction casting with ∼10^3^ K s^−1^ cooling rate [21]. In particular, we chose the Co‐Cr‐Fe‐Ni‐Ta system [22] as our model alloy since the Laves‐forming refractory element (Ta) directly facilitate the formation of significant modulus mismatch with the FCC matrix, while enabling nanoscale phase refinement through suction casting. (ii) engineering a hierarchical multiphase structure to reduce this hardness/modulus mismatch through further thermal treatment at a relatively low temperature of 600°C. Analogous to the biomineralization process, hierarchical multiple phases composed of FCC‐Laves eutectic lamellae with L1_2_ and D0_22_ coprecipitations, synergistically coordinating sessile defects, integrate crystallographic kinetics, topological defect, and interfacial engineering. This EHEA shows a near‐theoretical yield strength and retains an attractive plasticity. The deformation and strengthening mechanisms due to the above‐mentioned alloy‐design principles of this EHEA are evidenced by the integrated experiment and theoretical calculations. The present work demonstrates the critical importance of achieving long‐range modulus/hardness compatibility while engineering short‐range structural heterogeneity for advanced dual‐ and multi‐phase alloy systems.

## 2 Results and Discussion

### 2.1 Mechanical Properties of Designed Alloys

**FIGURE 1:**
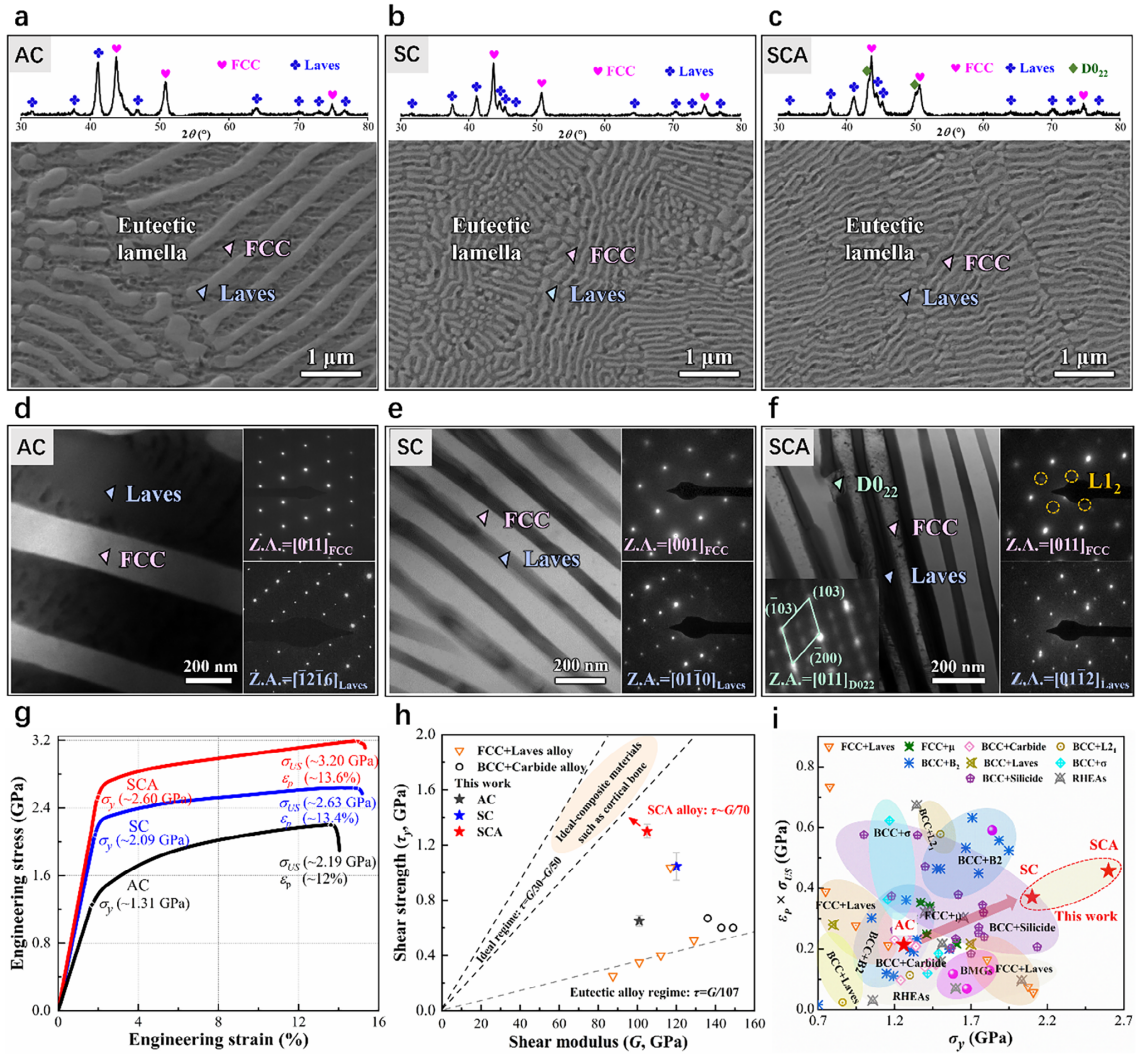
Refined nanolamellar structure and compressive mechanical properties of AC, SC, and SCA alloys. (a–c) Phase identification via XRD analysis and SEM images. (d–f) TEM images and the corresponding SAED patterns of eutectic lamellae (FCC and Laves), D0_22_ and L1_2_ precipitates. (g) Engineering stress–strain curves. (h) Shear strength, τ_*y*_ = σ_*y*_/2, plotted against shear modulus (*G*) for the SCA alloy and other dual‐phase alloy [23, 24]. Error bars denote the standard deviations of the strength values. (i) The products of plasticity and ultimate stress vs. yield stress of the AC, SC, and SCA alloys compared with the compressive mechanical properties of reported BCC + Laves, FCC + Laves, FCC + *µ*, BCC + Carbide, BCC + L2_1_, BCC + B2, BCC + *σ*, BCC + Silicide alloys, bulk metallic glasses (BMGs), and refractory high‐entropy alloys (RHEAs). The corresponding references in (i) are summarized in Table S2.

Our EHEA has a nominal composition of CoCrFeNiTa_0.4_ (at.%) and was fabricated using conventional arc melting (AC (as cast) alloy) followed by suction casting (SC alloy). Subsequently, the samples underwent an aging treatment at 600°C for 24 h (SCA alloy). We examined the microstructure evolution and its effect on yield‐strength enhancement in the three samples corresponding to the AC, SC, and SCA alloys, respectively. All three alloys exhibit a eutectic microstructure consisting of lamellar FCC and Laves phases (Figure 1a–c), while the SCA sample additionally contains a D0_22_ phase (Figure 1c). Compared to the AC alloy, suction casting significantly reduces the average lamellar thickness in the SC and SCA alloys. Specifically, the FCC lamellae reduce from 289 ± 6 nm (AC) to 84 ± 3 nm (SC) and 78 ± 1 nm (SCA), while the Laves lamellae decrease from 174 ± 5 nm (AC) to 52 ± 1 nm (SC) and 43 ± 1 nm (SCA) (Figures S2 and S3). Furthermore, unlike the AC and SC alloys containing only two lamellar phases (Figure 1), the SCA alloy has a hierarchical multiphase structure, containing soft‐hard inter‐assembled precipitations that the deformable D0_22_ precipitates within the hard Laves lamellae and the hard L1_2_ coherent precipitates within the soft FCC lamellae (Figure 1f).

To evaluate the mechanical performance of three alloys, we conducted compression tests at room temperature. Reducing the lamellar width significantly increased the yield strength from 1.3 GPa in the AC alloy to 2.1 GPa in the SC alloy, while maintaining good plasticity of 13.4% (Figure 1g). Further benefiting from the aging treatment, the SCA specimen achieved a superior combination of strength and plasticity. Specifically, the yield strength (σ_*y*_) and plastic strain (ε_*p*_) are 2.6 GPa and 13.6%, respectively (Figure 1g). Figure 1h shows the ultrahigh shear strength of the SCA alloy compared with other eutectic alloys. The shear stress (τ_*y*_) for the initiation of the plastic flow can be converted using τ_*y*_ = σ_*y*_/2 , a relationship suggested for compression experiments on alloys [25]. The shear modulus (*G*) of the SCA alloy is determined to be ∼95 GPa based on the nanoindentation experimentally extracted modulus. Notably, the τ_*y*_/*G* ratio of the SCA alloy reaches ∼1/70 (Figure 1h), which surpasses those of conventional eutectic alloys (τ_*y*_/*G* ∼ 1/107) [23, 24, 26] and approaches the theoretical shear strength limit (1/30 ∼ 1/50) [9]. To compare the mechanical performance of our EHEA with other dual‐phase alloys, we present a map of the product of ε_*p*_ and ultimate strength (σ_*US*_) vs. σ_*y*_ in Figure 1i. The product of ε_*p*_ and σ_*US*_ of the SCA specimen is comparable to that of BCC‐based dual‐phase alloys, but the yield strength of the SCA alloy is even 600 MPa higher than that of the strongest silicide‐strengthened BCC alloy. These results reveal that ultrastrong EHEAs can be realized by dual mechanisms involving lamellar refinement‐enhanced interfacial barriers and hierarchical multiphase structure‐induced hardness/modulus gradient minimization.

### 2.2 Hierarchical Multiple Phases

**FIGURE 2:**
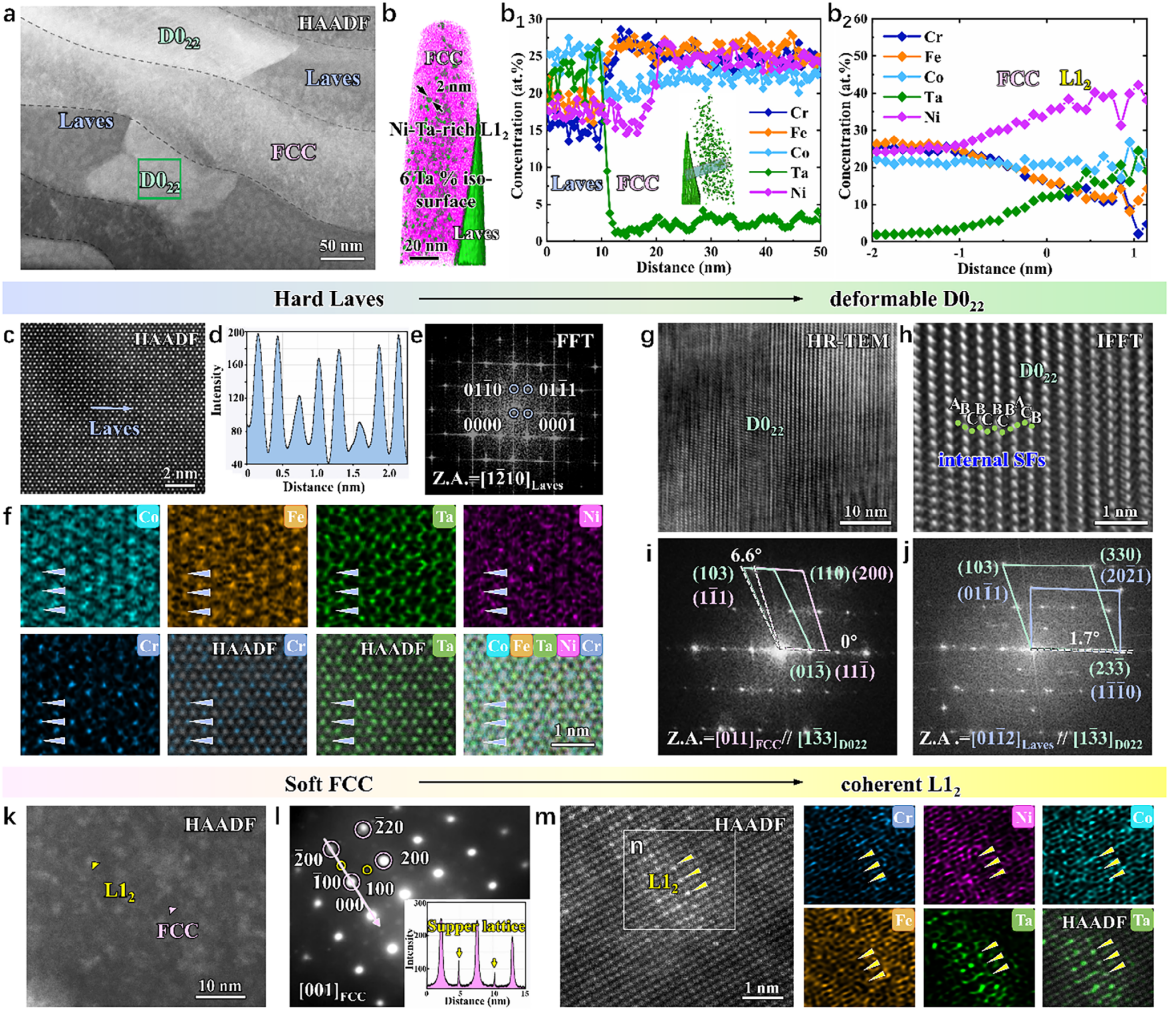
Hierarchical multiple phases of the SCA alloy. (a) The HAADF image of eutectic lamellae. (b) APT reconstruction and proximity histogram of 6 at.% Ta iso‐concentration surfaces presenting the morphologies and compositions of (b_1_) the FCC, Laves lamellae, and (b_2_) L1_2_ particles (∼2 nm). (c) The atomic‐resolution HAADF image of hard Laves lamellae. (d) The intensity profile along the blue arrow in (c,e,f) FFT patterns, and EDS mapping of Laves structure. (g) HRTEM image of D0_22_ precipitate taken from the [1$\bar{3}$3] zone axis, (h) an enlarged IFFT view of interior SFs within D0_22_ precipitate. (i,j) FFT patterns of FCC‐D0_22_ and Laves‐D0_22_ interfaces. (k–m) The HAADF, SAED patterns, and atomic‐resolution HAADF images show the morphology and atomic arrangement of L1_2_ precipitates. (n) Atomic‐resolution EDS mapping reveals the sublattice occupations of Cr (blue), Ni (purple), Co (blue‐green), Fe (orange), and Ta (green) atoms.

To explain the origin of the exceptional yield strength of the SCA specimen, we conducted an in‐depth analysis of its unique microstructure. Figure 2a shows that AB_2_ Laves lamellae partially transformed into the D0_22_ phase (known as the gamma double prime phase, *γ″*), an A_3_B‐intermetallic compound in Ni‐based superalloys [27, 28, 29]. No other phases or clusters were detected besides this D0_22_ phase (Figure S4). Elemental‐distribution mapping (Figure S5) reveals the partitioning behaviors of elements among the D0_22_, Laves, and FCC phases. Co is relatively uniformly distributed throughout all phases. Ta tends to partition preferentially into the D0_22_ and Laves phases, Ni prefers partitioning into the D0_22_ and FCC phases, while Fe and Cr exhibit a higher tendency to partition into the FCC lamellae.

Moreover, atom probe tomography (APT) characterization reveals the presence of L1_2_ precipitates with a volume fraction of 4.7% in the FCC lamellae (Figure 2b). These precipitates are absent in the SC (Figure S6) or AC alloys (Figure S7). Additionally, an L1_2_ depletion zone exists due to the high concentration of Ta partitioning into the phase boundary (Figure 2b_1_). The elemental concentration data are further used to calculate the volume fraction of the Laves phase using the lever rule: $f_{Laves}=\left(C_{FCC}^{i}-C_{0}^{i}\right)/\left(C_{FCC}^{i}-C_{Laves}^{i}\right)$, where $C_{0}^{i}$ is the nominal concentration of element, *i*, in the alloy, and $C_{j}^{i}$ represents the concentration of the same element in phase, *j*. By substituting the Ta‐concentration data into this equation, we determined that the Laves phase has a volume fraction of 28%.

The Laves lamellae are strongly chemically ordered, as evidenced by periodic brightness variations in the atomic‐resolution high‐angle annular dark‐field (HAADF) image and its intensity profile (Figure 2c,d), indicating that different atomic species preferentially occupy distinct lattice sites. Note that the brightness of each atomic column is roughly proportional to the square of the average atomic number (*Z^2^*) of column [30]. This chemically ordered Laves phase has a close‐packed hexagonal (HCP) crystal structure (Figure 2e). Atomic‐resolution EDS confirms that Ta atoms occupy the brighter columns, Cr atoms fill the dimmer ones, and lighter Co, Fe, and Ni atoms partially favor the Cr sublattice (Figure 2f). The Ta concentration is 22% (Table S1) for the stoichiometric composition of AB_2_ (TaCo_2_‐type) in the Laves phase. The particularly abundant occupancy of the Ta element among these elements provides the necessary conditions for D0_22_ transformations during aging. Ta provides the thermal driving force for D0_22_ precipitation through its highly negative mixing enthalpy with Ni (−29 kJ mol^−1^), while its segregation at phase boundaries reduces the nucleation barrier and its large atomic radius stabilizes the crystalline structure of the D0_22_ phase.

The D0_22_ phase exhibits a distinct atomic plane‐packing structure (Figure 2g) and possesses a tetragonal crystal structure. The observed faulted atomic‐plane stacking order in the HR‐TEM image (Figure 2h) corresponds to streaking lines perpendicular to the atomic planes in Figure 2i, indicative of stacking faults (SFs). Stacking faults indicate the defect‐mediated slip in the D0_22_ phase, demonstrating greater deformability than hard Laves lamellae. In addition, since the Ta concentration determined by EDS is only 13% (Table S1), the total concentration of Ni, Co, Fe, and Cr at the Ta sublattice sites must reach at least 12% to form the stoichiometric composition of A_3_B (Ni_3_Ta‐type). This feature suggests a low chemical ordering in the D0_22_ phase along with the lattice‐distortion effects [14, 31], providing additional evidence for the deformability of the D0_22_ precipitates. In Ni‐based superalloys, the *γ″* phase exists as coherent precipitates with an orientation relationship of (001)*_γ″_*//(001)_FCC_ and [100]*_γ″_*//[100]_FCC_ [32]. However, in the SCA specimen, the orientation relationship between the D0_22_ and FCC phases is ($01\bar{3}$)_D022_//($11\bar{1}$)_FCC_ and [1$\bar{3}$3]_D022_//[011]_FCC_ (Figure 2i), due to the crystal structure constraints during the transformation of the Laves phase into the D0_22_ phase. The orientation relationship between the Laves and D0_22_ phases is approximately ($1\bar{1}\bar{1}0$)_Laves_//($23\bar{3}$)_D022_ and [01$\bar{1}$2]_Laves_//[1$\bar{3}$3]_D022_ (Figure 2j), with a slight misalignment of ∼1.7° (Figure 2j). These advantageous interfacial characteristics are indispensable conditions for the D0_22_ transformation to take place.

The HAADF image of the FCC lamellae (Figure 2k) shows bright nanosized regions, which are identified as the ordered L1_2_ phase based on superlattice‐diffraction spots (Figure 2l). Consistently, atomic‐resolution EDS analyses (Figure 2m,n) reveal that the heavier Ta atoms occupy the brighter atomic columns, whereas the lighter Ni, Co, Cr, and Fe atoms mainly occupy the darker atomic columns. The Ta concentration, 18%, is lower than the 25% required for the A_3_B (Ni_3_Ta‐type) stoichiometry of the L1_2_ phase (Figure 2b_2_), indicating that Ni, Co, Cr, and Fe atoms with a combined concentration of 7% must occupy the Ta‐sublattice sites.

### 2.3 Sessile Defect Structures

**FIGURE 3:**
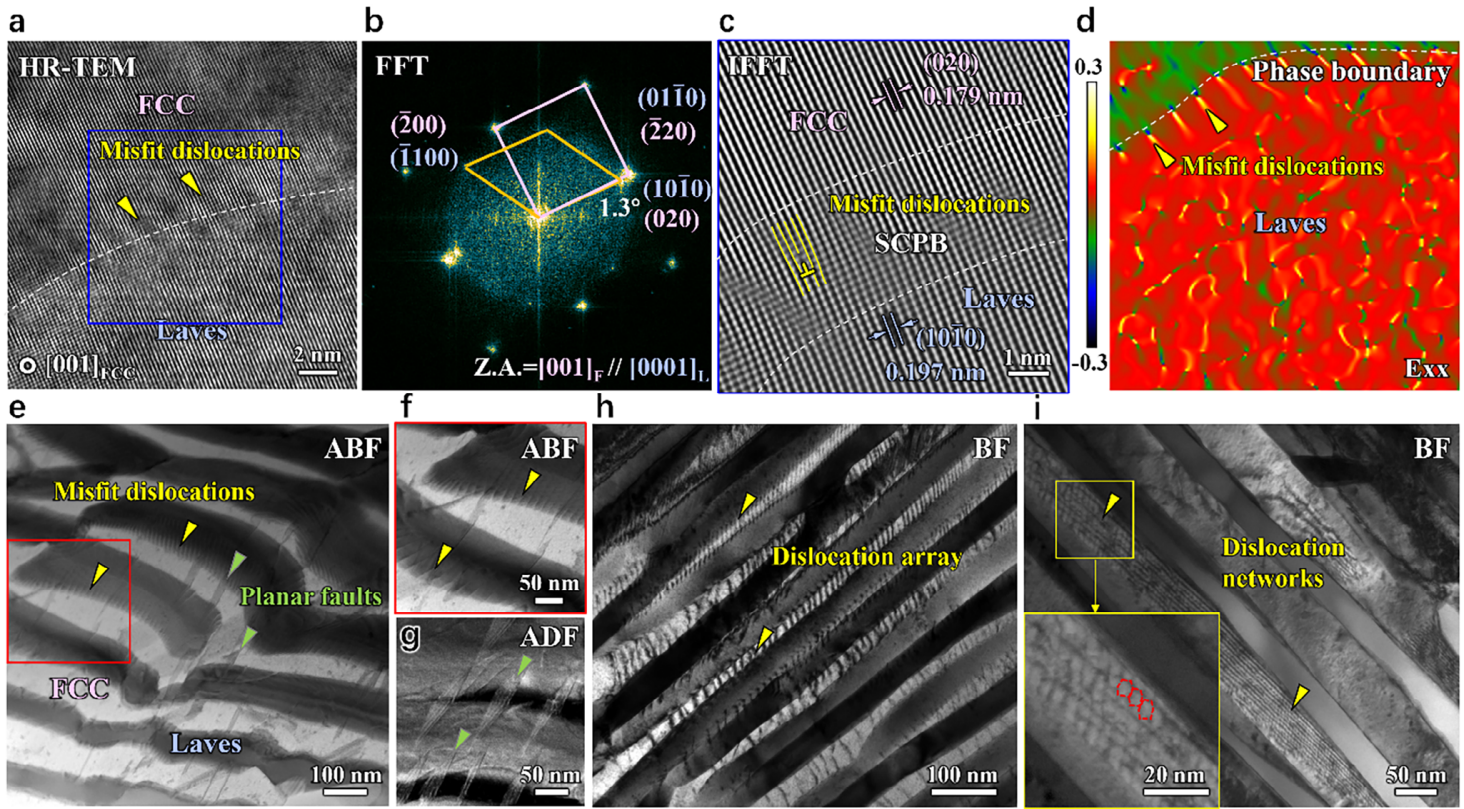
Sessile defects of the undeformed SCA alloy. (a,b) HR‐TEM image and corresponding FFT patterns of the FCC‐Laves interface. Accordingly, the misfit degree factor of the dual‐phase structure can be assessed. (c) A magnified IFFT image of the blue box in (a). (d) A GPA strain (horizontal normal strain, *ε_xx_*) map of (020)_FCC_//(10$\bar{1}$0)_Laves_ interface, showing misfit dislocations with a sharp strain gradient. (e) The annular bright‐field (ABF) STEM image of the dual‐phase interface feature. (f) The amplification of misfit dislocations. (g) The annular dark‐field (ADF) STEM image showing planar faults. (h,i) The dislocation array/networks formed within the FCC lamellae.

The FCC lamellae are also decorated with unique defect structures formed during suction casting and aging. In multiphase alloys, dislocations are generated in the soft phase after solidification due to internal stresses arising from differences in the mechanical properties (such as hardness and modulus [33, 34]) and thermal properties (such as solidification temperatures [35] and coefficients of thermal expansion [36]) among the phases. However, in the SCA alloy, the defect structure in the FCC lamellae is unique due to the crystallographic orientation correlation between the FCC and Laves phases. In particular, a semi‐coherent interface is observed between the FCC and Laves phases (Figure 3a,b). The orientation relationship between the FCC and Laves phases is (020)_FCC_//(10$\bar{1}$0)_Laves_ and [001]_FCC_//[0001]_Laves_. A corresponding mismatching degree factor ($\bar{\delta_{{\left(hkl\right)}_{n}}^{{\left(hkl\right)}_{s}}}$) is calculated based on the Bramfitt lattice matching theory [37]:

(1) $$\bar{\delta_{{\left(hkl\right)}_{n}}^{{\left(hkl\right)}_{s}}}=\sum_{i=1}^{3}\frac{\frac{\left|d_{{\left[uvw\right]}_{s}^{i}}cos\theta -d_{{\left[uvw\right]}_{n}^{i}}\right|}{d_{{\left[uvw\right]}_{n}^{i}}}}{3}\times \;100\%$$

where (hkl)_s_ and (hkl)_n_ are miller indices for the crystal planes of two phases, [uvw]_s_ and [uvw]_n_ are miller indices for the crystal directions on (hkl)_s_ and (hkl)_n_ planes, respectively, d_[uvw]s_ and d_[uvw]n_ are the interatomic spacing along [uvw]_s_ and [uvw]_n_, which are derived from the lattice parameters measured by XRD, *θ* is the included angle between two crystal planes, and its value is labeled in the pictures. The (020) plane spacing of the FCC phase is 0.179 nm, significantly smaller than the (10$\bar{1}$0) plane spacing of the Laves phase, which is 0.197 nm (Figure 3c), resulting in a large misfit factor of 9%. To accommodate this substantial lattice mismatch, interfacial dislocations are generated to release the misfit strain [38, 39, 40]. Geometrical phase analysis (GPA, Figure 3d) of the HR‐TEM image in Figure 3a reveals a twofold symmetrical elastic strain field around each misfit dislocation, arranged in a periodic array. These misfit dislocations slip along the {010}<100> system (Figure S9), rather than the conventional {111}<110> slip system in FCC crystals [41, 42]. Despite theoretical feasibility, this unconventional slip is inherently difficult.

Besides the periodic misfit dislocations prevailing at the interfaces of FCC and Laves lamellae (Figure 3e,f), defects terminating at adjacent interfaces are also formed within the soft FCC lamellae (Figure 3e). These planar faults are confirmed to be SFs through characteristic fringe contrast [43, 44, 45] (Figure 3g; Figure S9), which are often observed in Ni‐based superalloys with low stacking‐fault energies [46]. Another prevalent defect structure, rarely observed in other EHEAs, is a dislocation array penetrating the FCC lamellae (Figure 3h). These dislocation arrays are not observed in the SC alloy (Figure S11), indicating that they have low‐energy dislocation structures induced by aging. Moreover, dislocation networks arrays of interconnected dislocations often observed in crept Ni‐based superalloys [47, 48] are found in the FCC matrix (Figure 3i). High interfacial stresses during aging cause dislocations to interact and react with each other [44], resulting in hexagonal and octagonal cells (an enlarged view of Figure 3i) facilitated by the conversion of <110> to <100> dislocations [44, 47]. These stress‐induced dislocation reactions redistribute the interfacial strain energy, delaying the onset of localized deformation at the FCC‐Laves interfaces. Equally, the resulting low‐energy dislocation networks, containing sessile segments, further strengthen the SCA alloy by limiting the movement of mobile dislocations.

### 2.4 Strengthening and Deformation Mechanisms

The hierarchical multiple phases and sessile defects significantly influence the mechanical behavior of the SCA alloy, providing yield strength that is typically unattainable in conventional materials. GB strengthening, dislocation strengthening, and precipitation strengthening are the main possible routes to increase the FCC strength. The Hall‐Petch relationship is used to estimate the lamellae‐size dependence of yield strength in dual‐phase nanolamellar materials [49]. Dislocations initially start to propagate inside the soft FCC phase and pile up against the phase interface. It was assumed to contain a phase interface that can be crossed by pileups of *n* dislocations, of length *L*  = λ/2 (dislocation source at the lamellar center, λ is the thickness of the FCC lamella). Since *L*  = *n*μ*b*/4τ , the interface strength, τ_*B*_, must verify that τ_*B*_ =  *n*τ  = 2τ^2^λ/μ*b* , where μ is the shear modulus and *b* is the Burgers vector. In general, the critical applied shear stress, τ, of the dislocation‐slip transmission through the interface i,s related to the Hall‐Petch equation according to [5]

(2) $$\tau ={\left(\;\tau_{B}\mu /2\lambda_{FCC}\right)}^{1/2}=k{\left(\lambda_{FCC}\right)}^{-1/2}$$

where *k*  =  (τ_*B*_μ/2)^1/2^, a proportionality factor of the Hall‐Petch law. The critical shear stress upon plastic yielding can be estimated as [50]

(3) $$\tau =\frac{n\mu b}{4L}$$

where *n*  = ∼4 is the number of dislocations crossing the same lamellae [50], μ  = 81 GPa is the shear modulus of the FCC phase [5], and *b* is the Burger vector length (0.255 nm). Hence, the critical applied shear stress, τ ∼0.5 GPa. Using the Taylor factor (*M*) of the FCC phase (3.06 [12]), we estimated the strengthening contribution by the nanolamellar interface of about 1.5 GPa, making an important contribution to the high yield strength of the SCA alloy.

The high dislocation density of the FCC lamellae also makes a non‐negligible contribution to yield strength. This strengthening effect is estimated via the Taylor‐type hardening formula [12]

(4) $$\Delta \sigma_{dis}=M\alpha \mu b\rho^{1/2}$$

where α is the constant (0.2 for the FCC metal), ρ represents the dislocation density (5.5 ×  10^14^ m^2^) [5]. Accordingly, the strengthening effect by pre‐existing dislocations gives a total increase in yield strength of 0.29 GPa.

The specific contribution strengthened by the L1_2_ precipitates contains

(5) $$\Delta \sigma_{CS}=M\alpha {\left(\mu \varepsilon \right)}^{3/2}\;{\left(\frac{rf}{0.5\mu b}\right)}^{1/2}$$

(6) $$\Delta \sigma_{MS}=M\cdot 0.0055\;{\left(\Delta \mu \right)}^{3/2}\;{\left(\frac{2f}{\mu }\right)}^{1/2}{\left(\frac{r}{b}\right)}^{3m/2-1}$$

(7) $$\Delta \sigma_{OS}=M\cdot 0.81\;\frac{\gamma_{APB}}{2b}{\left(\frac{3\pi f}{8}\right)}^{1/2}$$

where Δμ is the shear‐modulus mismatch between the matrix and precipitates, ε is the constrain lattice‐parameter mismatch between the L1_2_ phase and FCC matrix (0.21% from the HR‐TEM image), the average particle diameter, *r*, is about 2 nm, and *f* is the volume fraction of L1_2_ precipitates (∼5% from APT), *m* is a constant taken to be 0.85 [12], γ_*APB*_ is the anti‐phase boundary energy of L1_2_ precipitates [12]. The calculated individual values of Δσ_*CS*_, Δσ_*MS*_ and Δσ_*OS*_ are 0.03, 0.02, and 0.14 GPa, respectively. The particle‐matrix coherency (Δσ_*CS*_) and modulus mismatch (Δσ_*MS*_) make contributions prior to shearing, while the atomic‐ordering strengthening (Δσ_*OS*_) contributes during shearing. In principle, the larger one of Δσ_*CS*_+ Δσ_*MS*_ and Δσ_*OS*_ determines the resultant contribution, indicating L1_2_‐precipitation strengthening of 0.14 GPa. Generally, the grain‐boundary strengthening increases from 0.50 GPa in the AC specimen to 1.54 GPa in the SC and SCA specimens by reducing the lamellar width. The L1_2_ precipitation and sessile defect synergistically enhance the FCC lamellae, resulting in the increases of yield strength for 0.14 and 0.29 GPa, respectively, which effectively mitigates the modulus/hardness mismatch between the FCC and Laves lamellae (Figure S12).

A total increase in the yield strength of the SCA alloy was estimated through the rule of mixture (ROM) of the respective contribution from FCC and Laves phases. For the ROM, the equal‐strain [51] assumption and equal‐stress [52] assumption have been widely employed. When the deformation of lamellae was governed by the isostrain condition, it requires the equal deformation of both phases [53]. The plastic deformation or buckling of the stronger Laves lamellae governs the yielding process, leading to the highest yield stress. While the overall yielding was dominated by the plasticity of the softer FCC lamellae that was controlled by the isostress condition, resulting in the lowest yield stress [54]. For orientations between 0° and 90°, the yield strength falls between the values predicted by the isostrain and isostress conditions. Hence, the equal strain treatment, which is an upper bound, and the equal stress treatment, which is a lower bound, can be used for the yield stress of the dual‐phase in situ composite

(8) $$\sigma^{upper}=V_{FCC}\;\sigma_{FCC}+V_{Laves}\sigma_{Laves}$$

(9) $$\frac{1}{\sigma^{lower}}=\frac{V_{FCC}}{\sigma_{FCC}}+\frac{V_{Laves}}{\sigma_{Laves}}$$

where *V_FCC_* (72%, calculated from the APT results) and *V_Laves_* (28%, calculated from the APT results) are the volume fractions of soft and hard phases, while σ_*FCC*_ (1.50 GPa + 0.29 GPa + 0.14 GPa, the aforementioned calculation outcomes) and σ_*Laves*_ (7.3 GPa [16]) are the strength of soft and hard phases, respectively. Therefore, the combined strengthening effects in the SCA alloy from the nanolamellar structure, sessile dislocations, and L1_2_ precipitates give a span of 2.5 GPa (lower bound) ∼3.5 GPa (upper bound), which ideally includes the measured yield strength. The lower bound corresponds to an iso‐stress [52] condition for a composite, while the upper bound corresponds to an iso‐strain [51] condition. It should be noted that the D0_22_ phase within the Laves lamellae was not considered in the yield‐strength estimation. The slightly overestimated yield strength range for the SCA specimen arises because the yield strength of the Laves lamellae should be decreased due to the partial transformation of the harder Laves phase into the softer D0_22_ phase. Nevertheless, the yield strength of the EHEA that we studied can be further improved by controlling the texture of the Lamellae.

**FIGURE 4:**
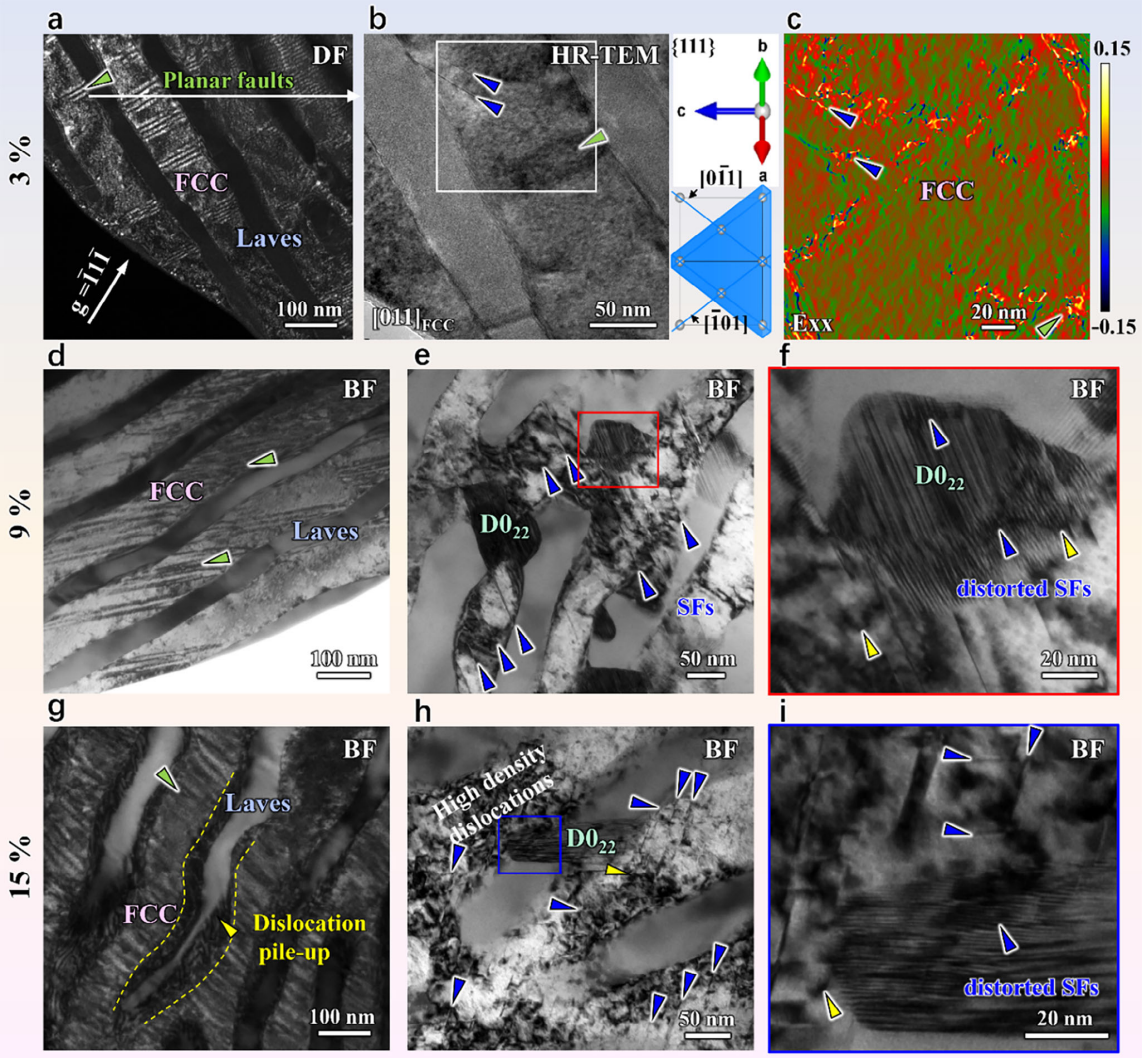
Deformation micro‐mechanisms of the SCA alloy. (a–c) Deformation microstructure at the 3% strain. (a) Dark field (DF) TEM image of sessile planar faults (green triangle) that divide FCC lamellae into small blocks. (b) Enlarged HR‐TEM of the region in (a), showing SFs and immature planar faults lying on the {111} plane. The (111) plane perpendicular to the [011] axis zone is represented by the blue plane on the right, and the directions of [0$\bar{1}$1] and [$\bar{1}$01] are expressed by blue lines. (c) GPA strain mapping. The positive strain corresponds to the SFs and planar faults. (d) Activations of more planar faults and interaction with dislocations at the 9% strain. (e,f) The distorted SFs in D0_22_ precipitates indicating a synergistically multiphase‐deformation mechanism. (g) Dislocations arranged at the phase interface, and the SFs restricted in the soft FCC lamellae at the fracture strain. (h,i) Severely deformed D0_22_ phase.

Despite its extremely high yield strength, the SCA alloy exhibits ductile behavior. To elucidate the underlying mechanism, we conducted a TEM analysis of deformation defects in the SCA alloy subjected to different strain levels (Figure 4). At a strain of 3%, the SCA alloy shows relatively uniform plastic deformation (Figure 4a) compared to the heterogeneous dislocation distribution observed in the SC and AC specimens (Figure S13). The dominant deformation mechanism in the FCC lamellae is dislocation slip with occasional SFs, while no plastic deformation occurs in the hard Laves lamellae (Figure 4b). The preexisting sessile SFs in the SCA alloy delay dislocation pileups at lamellar interfaces, as evidenced by the relatively high elastic strain around SFs (Figure 4c). When the strain increases to 9%, dislocation pile‐up at lamellar interfaces is still less evident, but dislocations distribute more heterogeneously within the FCC lamellae (Figure 4d). Meanwhile, the density of SFs increases (Figure 4d,e), further dividing the FCC lamellae into nanoscale blocks, approximately with 20 nm width, providing a dynamic Hall‐Petch strengthening effect [55, 56, 57]. Additionally, the D0_22_ precipitates coherent with the FCC lamellae begin to deform, as evidenced by the orientation change of SFs (Figure 4f). These D0_22_ precipitates facilitate load transfer within the soft FCC lamellae and alleviate stress concentrations, thereby delaying crack initiation. Upon fracture of the SCA alloy, a high density of dislocations accumulates near phase boundaries and within the FCC phase, and the Laves lamellae begin to twist (Figure 4g). Besides, more SFs form within the FCC matrix (Figure 4h). The preexisting SFs in the D0_22_ phases become highly distorted with multiple orientation changes, effectively alleviating stress concentration at the interface and coordinating deformation between the two phases. Consequently, the hierarchical multiphase structure and sessile defects in the SCA alloy enable a hardness/modulus match, promoting coordinated deformation of the eutectic phases. This synergistic effect significantly enhances mechanical performance, achieving superior strength‐ductility synergy compared to conventional materials.

## 3 Conclusion

In summary, we have developed a class of eutectic alloys through suction casting and post‐treatment process, achieving nanometer‐thick lamellae and hardness/modulus matching driven by hierarchical multiple phases and sessile defects. The SCA alloy exhibits near‐theoretical yield strength and impressive plasticity, outperforming other state‐of‐the‐art alloys. The modified multiphase microstructure plays a critical role in strengthening interfaces, mediating strain delocalization, and activating multiple‐deformation mechanisms. The strengthening‐toughening mechanisms derived from structural refinement and hardness/modulus match can be applied to the design of high‐performance multiphase alloys.

## 4 Experimental Methods

### 4.1 Materials Preparation

The EHEA ingots with a nominal composition of CoCrFeNiTa_0.4_ (at.%) were synthesized using vacuum arc melting under an argon atmosphere. Elemental Co, Cr, Fe, Ni, and Ta, each with a purity of at least 99.9 wt.%, were used as raw materials. Each ingot (denoted as the as‐cast (AC) alloy) was melted five times to ensure compositional uniformity. Some ingots were suction‐casted into a water‐cooled copper mold with dimensions of *Φ* 3 mm × 60 mm to produce rods labeled as the SC (suction‐cast) alloy. Some SC rods were then aged at 600°C for 24 h (Text S1) and labeled as the SCA (suction‐casting aged) alloy. Cylindrical samples with dimensions of *Φ* 3 mm × 5 mm were prepared for mechanical tests via wire‐electrode cutting from the AC ingots, SC rods, and SCA rods. The cooling rate ($\dot{T}$) realized in a cylindrical rod sample with a typical dimension of *R*, during the copper mold casting, can be estimated as follows: $\dot{T}$= 10/*R* ^2^ [58], Where, the units of $\dot{T}$ and *R* are K s^−1^ and cm, respectively. Consequently, the rate of cooling of the SCA alloy (d = 3 mm) was estimated to be 4.4 × 10^2^ K s^−1^. Whereas, the cooling rate in AC alloy was determined to be ∼2.5 K s^−1^ [59]. For the preparation of TEM samples at different strain levels, two interrupted tests were performed on strains of 3% and 9% on three EHEA specimens.

### 4.2 Mechanical Tests

At room temperature, compression tests were conducted using an Instron 5982 universal testing machine at a strain rate of 1 × 10^−3^ s^−1^. Before the test, the ends of all samples were polished down to 2000‐grit using SiC paper. To ensure accuracy, three samples were tested under each condition. Nanoindentation tests of AC, SC, and SCA alloys were conducted using the NanoTest System (Micro Materials Ltd.). A 7 × 7 array was indented with an interval of 3 µm on the polished surface of each sample. Both the loading and unloading rates were 0.25 mN s^−1^, and the maximum indentation load was 5 mN with a dwell time of 10 s.

### 4.3 Structure and Composition Characterization

An X‐ray diffractometer (XRD, Bruker D8‐Discover) with Cu‐Kα radiation was used to measure the phase structures of the EHEAs at a scanning rate of 0.5° min^−1^. A field‐emission scanning electron microscope (SEM, Zeiss Crossbeam350) was employed to characterize the microstructures. A field‐emission transmission electron microscope (TEM, Thermofisher F200X) and an aberration‐corrected scanning transmission electron microscope (STEM, Themis Spectra 300) with spatial resolutions up to 60 pm were used to characterize the atomic structures and atomic‐resolution chemical composition. HAADF images were recorded at 300 kV. For the TEM observation, specimens were first mechanically ground to 60 µm, followed by punching into discs with a diameter of 3 mm, and then thinned by ion‐milling (GATAN‐M691). The volume fraction of the D0_22_ precipitates was measured from more than TEM images.

Atom probe tomography (APT) and 3D elemental distribution analyses were performed in the CAMECA Instruments LEAP 4000X Si local electrode atom‐probe system. The specimens were analyzed in a laser‐pulse mode under an ultrahigh vacuum of approximately 2.5 × 10^−11^ Torr at 40 K, a pulse repetition rate of 200 kHz, and a UV laser energy of 60 pJ at a 20% pulse fraction. Sharp tip specimens for the APT tests were prepared by focused ion beam milling on a dual‐beam Zeiss Auriga. The CAMECA integrated visualization and analysis software IVAS, version 3.8.2 [60], was used for data processing and 3D atomic reconstruction.

## Author Contributions

Yusha Luo: Conceptualization, Data curation, Formal analysis, Methodology, Visualization, Writing – original draft. Qianqian Wang: Investigation, Supervision, Writing – review and editing. Bo Sun: Conceptualization, Software, Validation. Ruixin Sheng: Data curation. Zhijun Guo: Data curation. Gaopeng Zou: Conceptualization. Zhe Jia: Validation, Resources, Writing – review and editing. Yang Tong: Data curation, Formal analysis, Resources, Writing – review and editing. Gang Sha: Data curation. Peter K. Liaw: Writing – review and editing. Baolong Shen: Funding acquisition, Resources, Supervision, Validation, Writing – review and editing.

## Conflicts of Interest

The authors declare no conflicts of interest.

## Supporting information




**Supporting File**: advs73615‐sup‐0001‐SuppMat.docx.

## Data Availability

Data will be made available on request.
