# Supplementary material for: Deformable Eutectic Alloy With Near‐Theoretical Yield Strength via Hierarchical Nanoscale Multiphases and Sessile Defects
Source: Adv Sci (Weinh). 2026 Jan 5;13(14):e18764. doi: 10.1002/advs.202518764 (PMC12970223; doi:10.1002/advs.202518764)
Supplement: Supplementary file 1 — Supporting File: advs73615‐sup‐0001‐SuppMat.docx. [file ADVS-13-e18764-s001.docx]

Supporting Information

Deformable Eutectic Alloy with Near-theoretical Yield Strength via Hierarchical Nanoscale Multiphases and Sessile Defects

Yusha Luo^1, 2^, Qianqian Wang^1, 2, *^, Bo Sun^1, 2^, Ruixin Sheng^1, 2^, Zhijun Guo^1, 2^, Gaopeng Zou^1, 2^, Zhe Jia^1, 2^, Yang Tong^3, *^, Gang Sha^4^, Peter K. Liaw^5^, Baolong Shen ^1, 2, *^

This PDF file includes:

Supplementary Text 1-4

Figure S1 to S14

Tables S1 to S3

References (61 to 95)

**Supplementary Text 1 | Selection of aging temperature and time**

Previous studies suggest that the effective aging temperature is near 0.5*T*_m_.^[61]^ The aging temperature in this work is 600 °C, which is equivalent to 0.46*T*_m_.^[62]^ As reported by Fan L.,^[63]^ the relatively low temperature of 600 °C leads to sluggish bulk diffusion. This kinetically suppresses the continuous precipitation of spherical L1_2_ nanoparticles in the FCC matrix, thereby preventing their coarse growth and encouraging a uniform distribution of beneficial nanoscale precipitates. Consequently, we selected the aging temperature of 600 ℃. After observing negligible L1_2_ precipitation following 6 and 12 h aging at 600 °C (**Figure S1a, b**), the treatment time was extended. Aging for 24 h produced fine L1_2_ precipitates, which, however, coarsened rapidly after 48 h (**Figure S1c, d**). Therefore, the treatment at 600 °C for 24 hours was chosen as it represents the condition that yields the most desirable microstructure for enhancing mechanical properties.


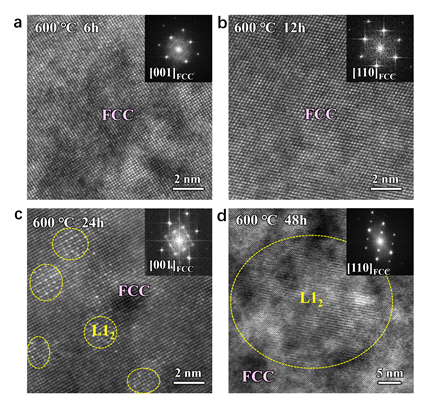


**Figure S1** HRTEM images and SAED patterns at the 600 ℃ aging for (a) 6 h, (b) 12 h, (c) 24h, and (d) 48 h.


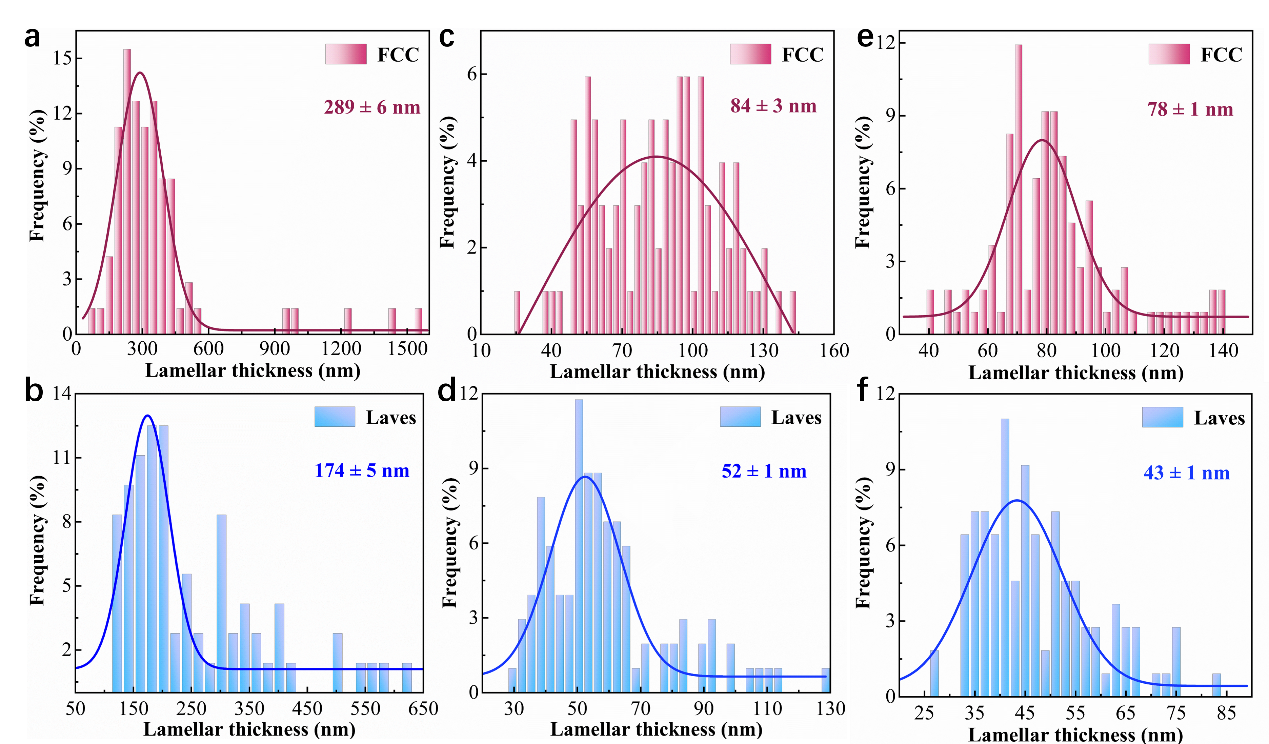


**Figure S2 Lamellar thickness distribution of the FCC and Laves phases in a-b AC, c-d SC, and e-f SCA specimens.** Suction casting has a faster cooling rate than arc melting, so SC and SCA alloys exhibit a refined nanolamellae structure.


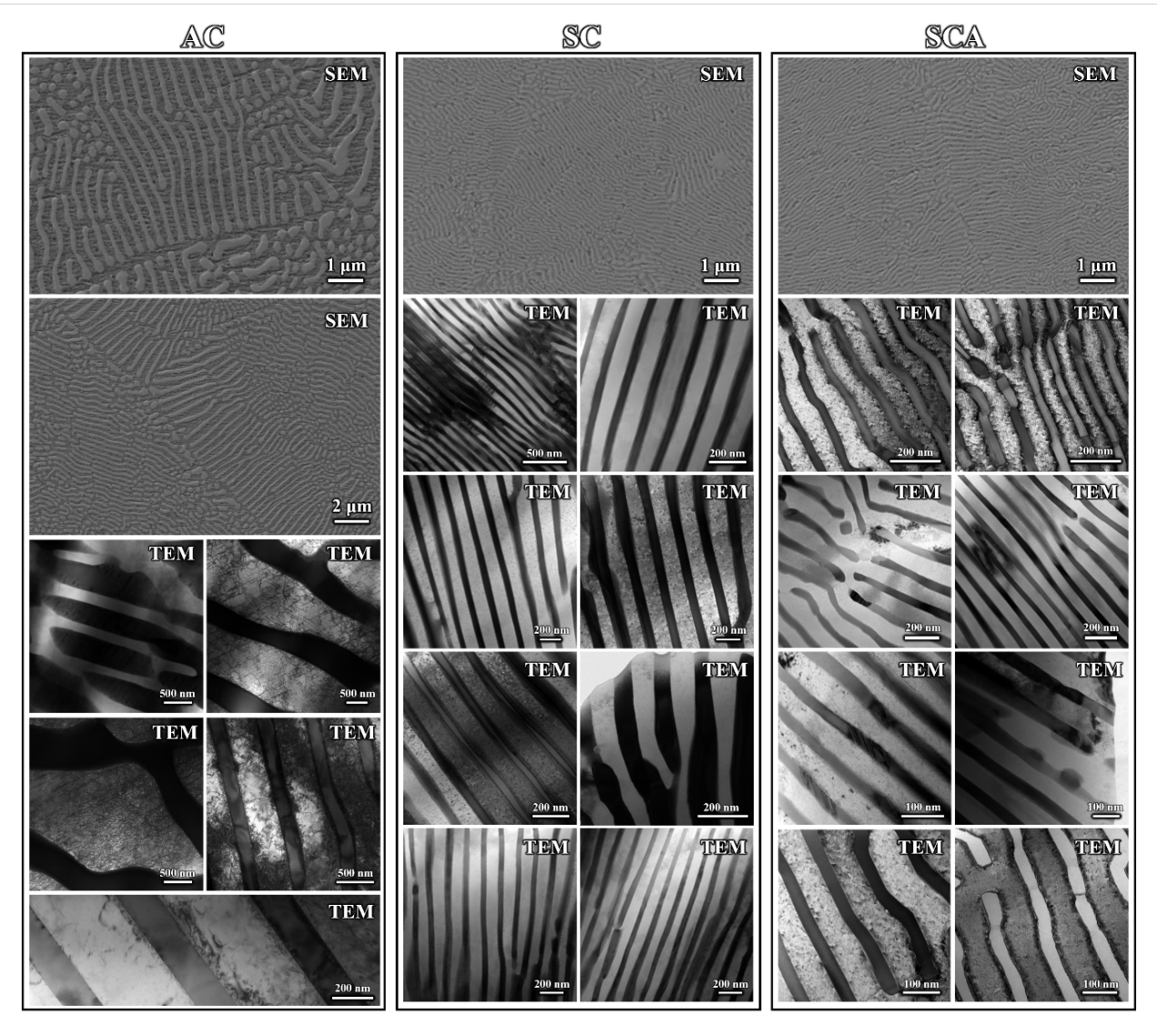


**Figure S3** Representative SEM and TEM micrographs used for the statistical analysis (**a**) AC

alloy, (b) SC alloy, and (**c**) SCA alloy.


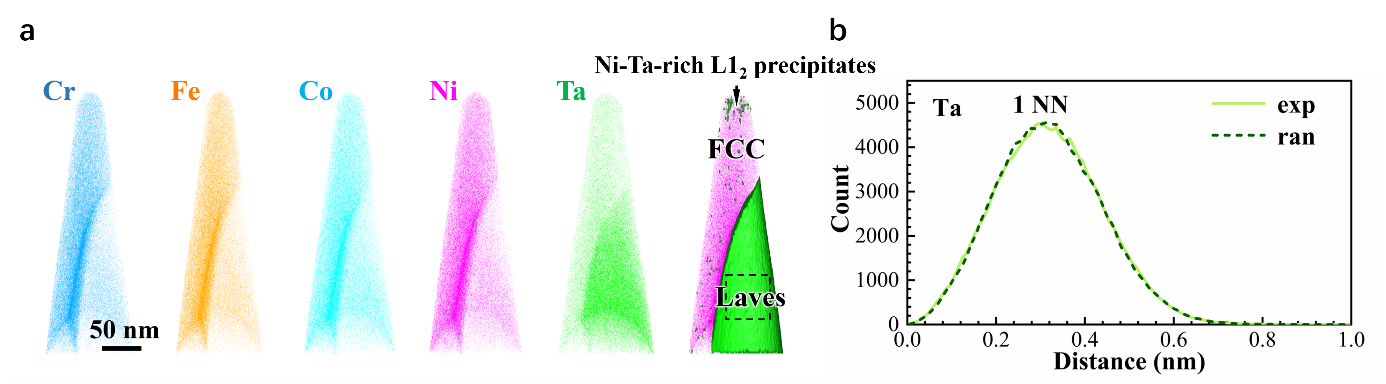


**Figure S4** **a** APT results present the compositions of the FCC and Laves lamellae in the SCA alloy; **b** The1-nearest neighbour distance analysis for Laves phase to confirm the possible cluster. A green-dotted line represents the random distribution curve of the elements, and the green-solid line represents the experimentally elemental distribution curve. The best fit between the experimental and theoretical curve indicates that the Ta atoms are randomly distributed without cluster. The strongly negative mixing enthalpies for Ta–Ni (-29 kJ·mol⁻^1^) and Ta–Co (-24 kJ·mol⁻^1^) promote the formation of Ta–Ni-rich L1_2_ and Ta–Co-rich Laves phases, respectively.


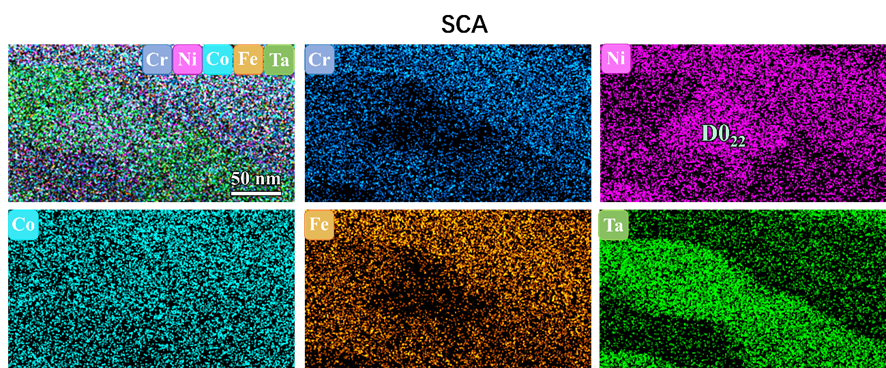


**Figure S5** EDS mapping of eutectic lamellae in the SCA alloys. The images show an accomplished transformation from a hard Laves structure to deformable D0_22_ phase, which enriches Ni and Ta elements, but lackes in Cr and Co.


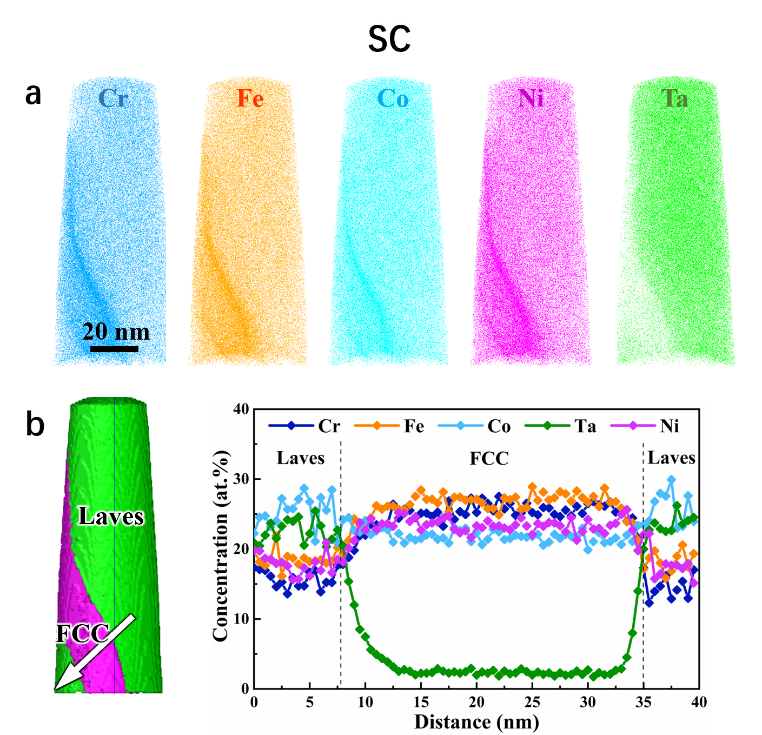


**Figure S6 APT characterization on the FCC and Laves lamellae in the SC alloy. a** 3D- reconstruction maps. **b** The 6 at% Ta iso-concentration surface shows the reference phase boundary. One-dimensional compositional profiles across the interface of the FCC/Laves phase. The 3D-reconstruction maps reveal that Fe, Ni, and Cr are partitioned into an FCC phase with an average composition of Co_22.03_Cr_25.36_Fe_26.93_Ni_23.34_Ta_2.34_, while Co and Ta are partitioned into the Laves phase with an average composition of Co_24.23_Cr_16.97_Fe_18.94_Ni_17.26_Ta_22.59_. No precipitate was detected in the FCC or Laves lamellae.


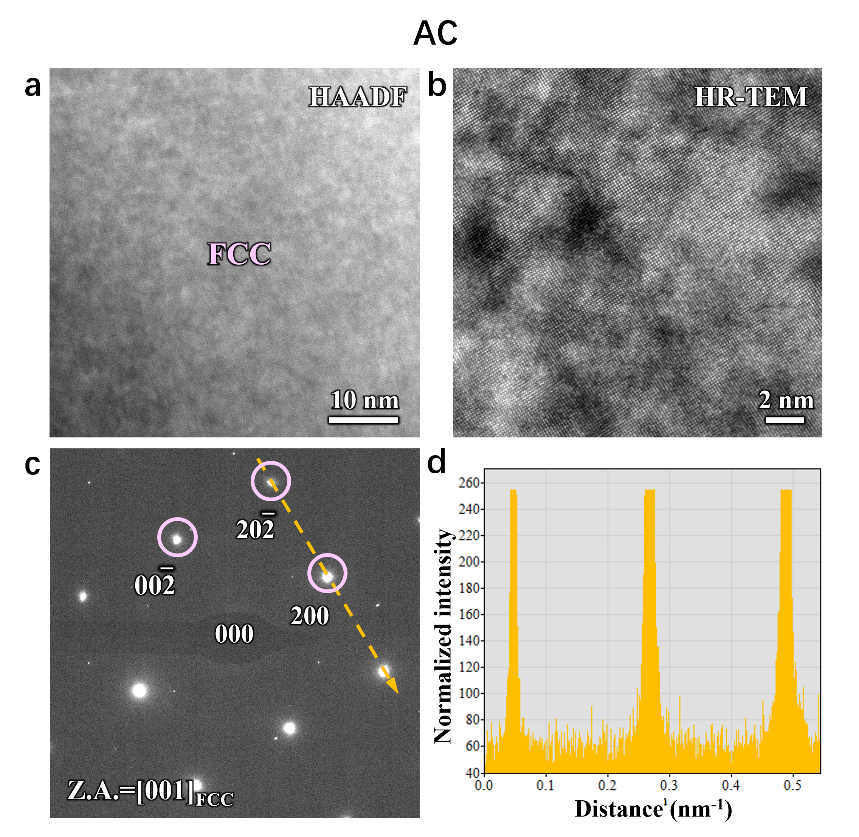


**Figure S7 Microstructure of the FCC lamellae in AC alloy. a** HAADF and **b** HR-TEM images, **c** SAED patterns, and **d** the intensity profiles along the yellow dashed arrow. No alternating intensity variation is observed in the SAED diffractogram along the [001] beam incidence, suggesting that no apparent ordered L1_2_ phase is present.


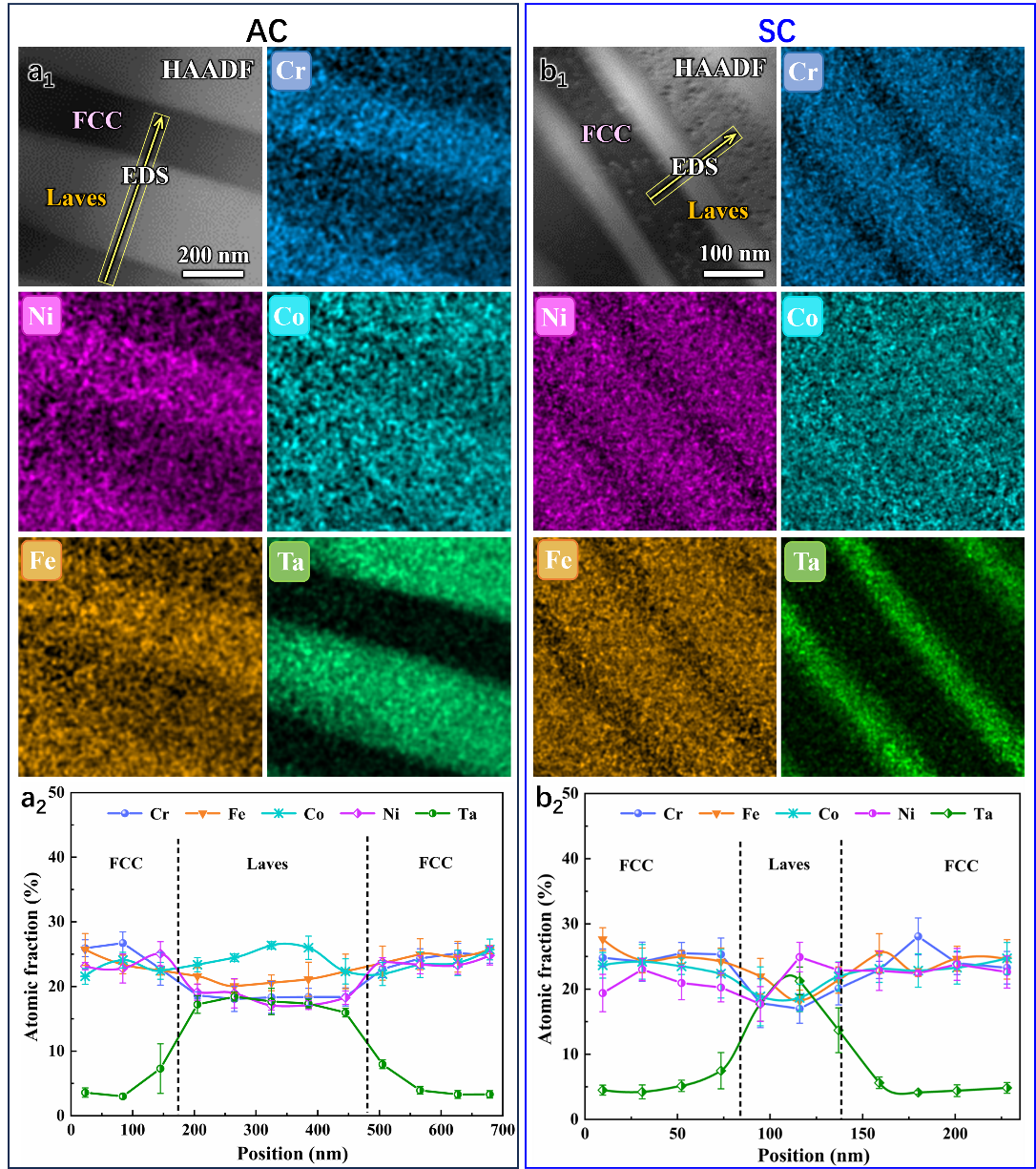


**Figure S8 Composition analyses of the AC and SC alloys.** HAADF images and EDS maps of **a_1_** AC and **b_1_** SC alloys show the distribution of Co, Cr, Fe, Ni, and Ta. The corresponding EDS composition profiles across FCC and Laves dual-phase lamellae marked in a_1_ and b_1_ are exhibited in **a_2_** and **b_2_**.


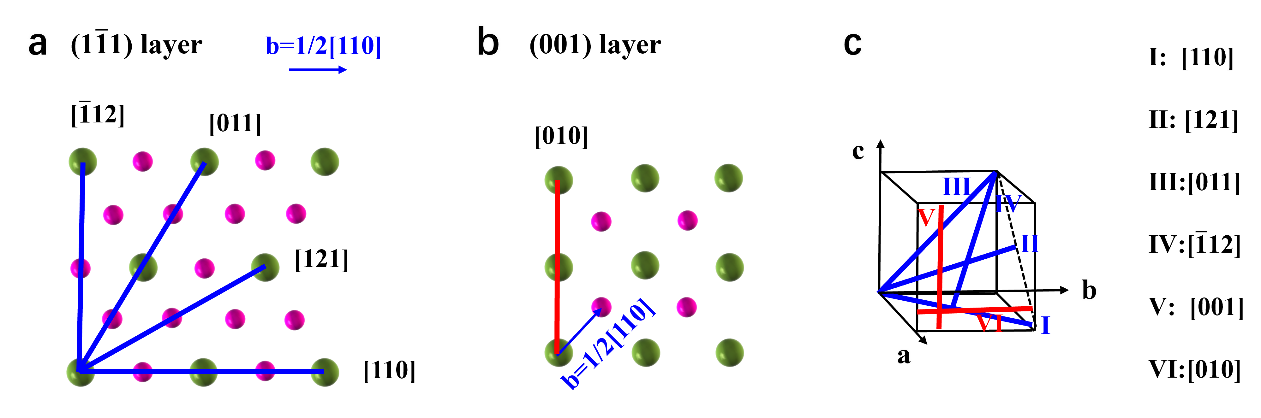


**Figure S9 Schematics of the mobile dislocations and misfit dislocations of the FCC lamellae in the SCA alloy. a** Mobile dislocations on the (1$\bar{\text{1}}$1) plane. **b** Misfit dislocations at the lamellar interface. **c** Spatial positions of the mobile and misfit dislocations in the FCC unit cell. Type Ⅰ-Ⅳ (blue) are the mobile dislocations, and Ⅴ-Ⅵ (red) are the misfit dislocations.

**Supplementary Text 2 | Planar defect in the SCA alloy**

As shown in Figure S10b, the FFT of the planar-fault zone exhibits a distinct L1_2_ superlattice pattern, indicating intense interactions between the planar faults and L1_2_ precipitates. In addition, the numerous dislocations slipping on the (111) plane within the planar-fault zone may potentially create partial dislocations across multiple contiguous L1_2_ precipitates, forming superlattice SF. As mentioned by Titus M. S.,^[64]^ superlattice intrinsic stacking faults (SISFs) contain a bright fringe on the side of the fault toward which the g-vector points, while superlattice extrinsic stacking faults (SESFs) contain the bright fringe on the other side of the fault, relative to the g-vector. As reported by previous studies, ^[64-68]^ the sessile nature of these planar fault substructures suggests they are superlattice extrinsic SF (SESF) or superlattice intrinsic SF (SISF),^[43, 69]^ which formed on a {111} plane by the glide of a 1/3<112>, enabling partial dislocations across multiple contiguous L1_2_ precipitates. The 1/3[121] dislocation enters the precipitate, leaving a superlattice intrinsic stacking fault (SISF) in its wake, while the 1/6[121] dislocation is left at the interface.^[65]^ The shearing process, which includes the reaction of dislocations at the FCC/L1_2_ full-coherent interfaces to form partial dislocations and gliding of the partial dislocations across the L1_2_ precipitates, is a thermally activated phenomenon in this alloy system.


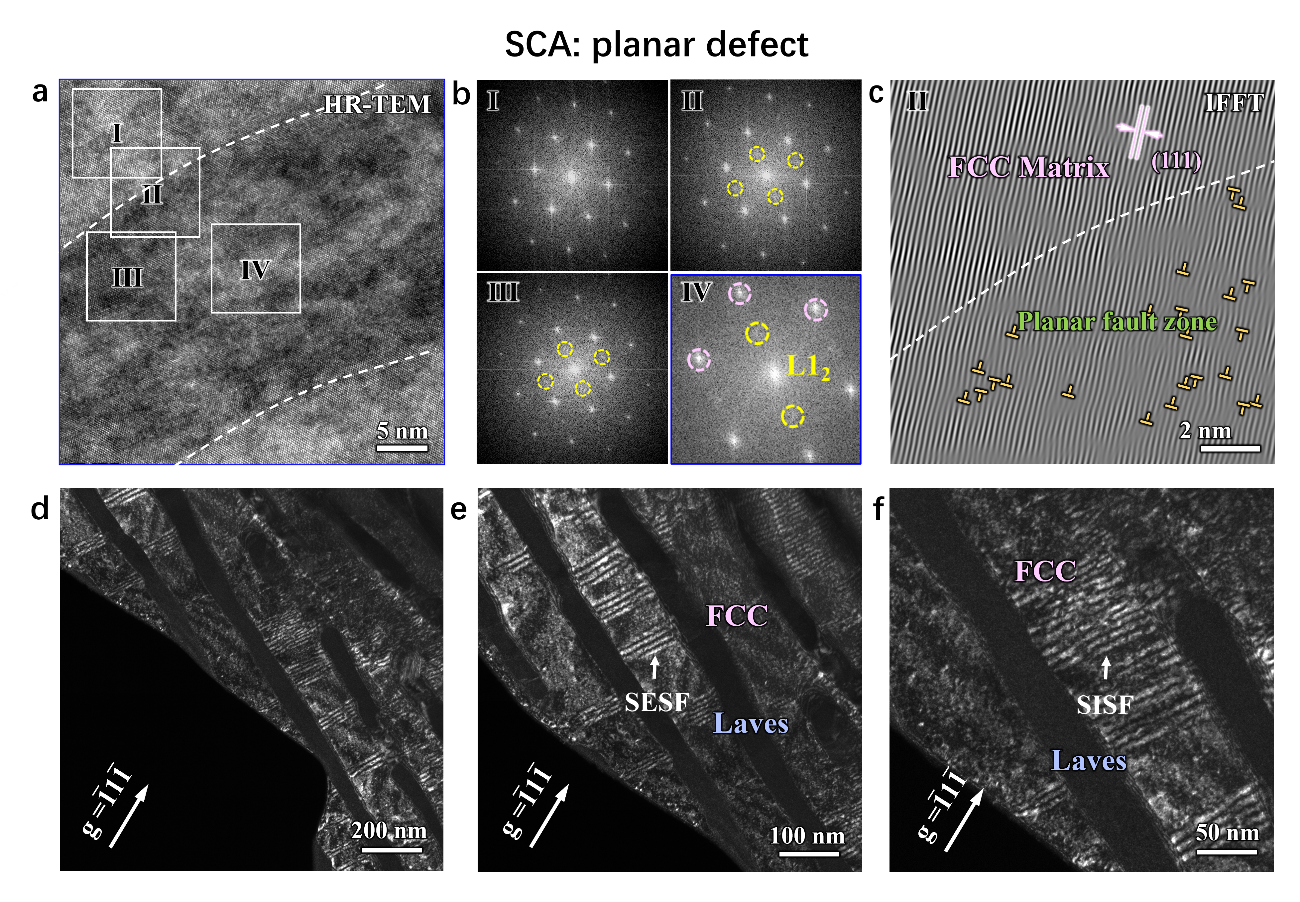


**Figure S10 Microstructure of the planar fault. a** HR-TEM of planar fault. **b** The FFT images corresponding to the regions of Ⅰ, Ⅱ, Ⅲ, and Ⅳ marked in **a**. **c** Inverse FFT (IFFT) image of region Ⅱ. **d-f** DF TEM images of sessile SFs (SISF or SESF) that divide FCC lamellae into small blocks.


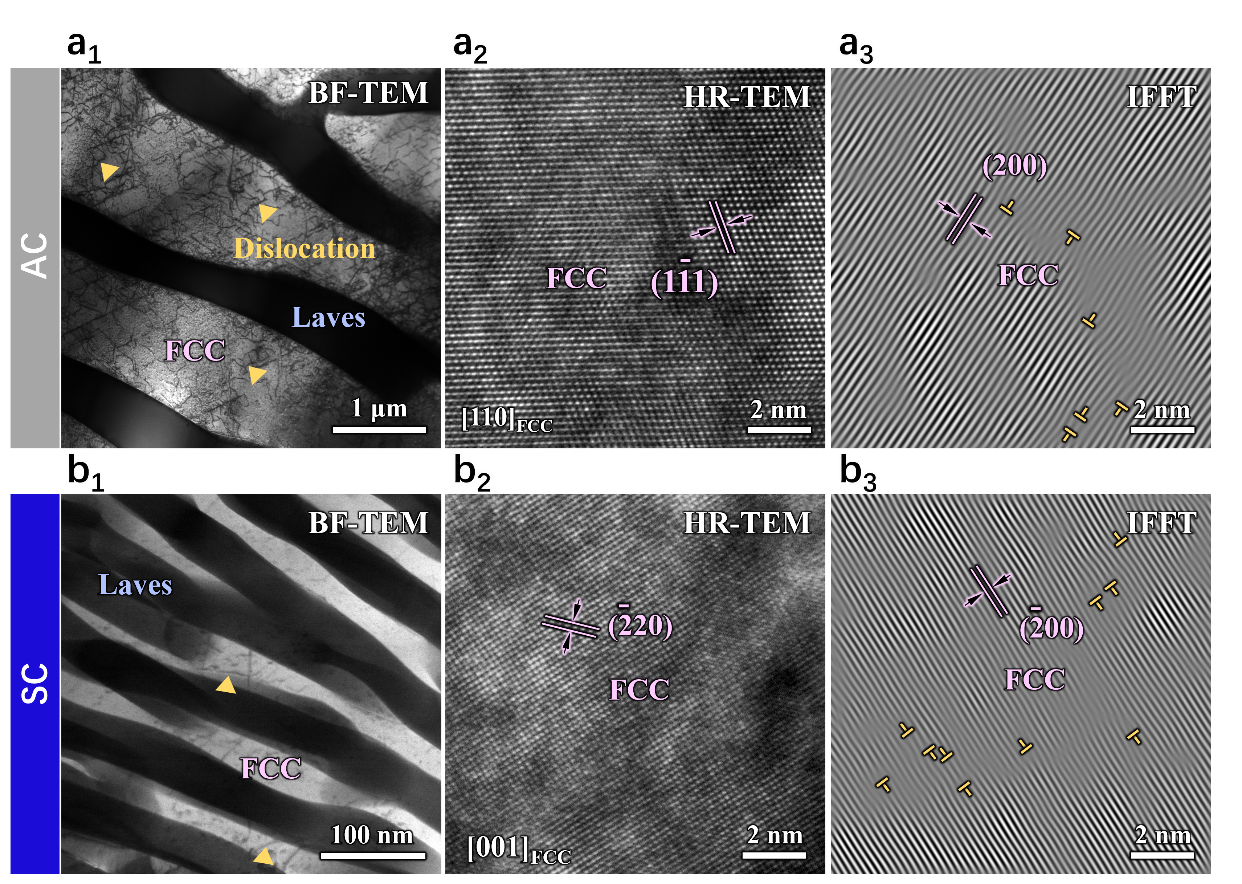


**Figure S11 The defect structure of the undeformed AC and SC alloys. a_1_-a_3_** BF-TEM, HR-TEM along the [110] axis zone and corresponding IFFT images in the AC alloy, **b_1_-b_3_** BF-TEM, HR-TEM along the [001] axis zone and corresponding IFFT images in the SC alloy. It is the dislocations that prevail before deformation in AC and SC alloys.

**Supplementary Text 3 | Comparisons of the modulus and hardness of three alloys**

It is challenging to perform nanoindentation on a single phase within a nanoscale lamella. Instead, both the soft FCC phase and hard Laves phase jointly bear the applied load (SEM images of **Figure S11, a-c**). The SCA alloy exhibits higher strength and ductility (**Figure 1f**) but slightly lower hardness and elastic modulus (**Figure S11, h and i**), indicating softened hard phases and hardened soft phases. Contour-line maps of modulus and hardness (**Figure S11, h and i**) showing a more uniform hardness distribution for the SCA alloy, also confirm the reduced modulus mismatch driven by coprecipitations and sessile defects. In addition, the shear modulus of alloys can be estimated via the reduced modulus, *E_r_*. By referencing the FCC modulus values reported in the literature and applying the rule of mixtures, the shear modulus of the Laves phase is inferred to be approximately 178 GPa. This trend provides a basis for estimating the theoretical strength of other FCC-Laves dual-phase alloys.


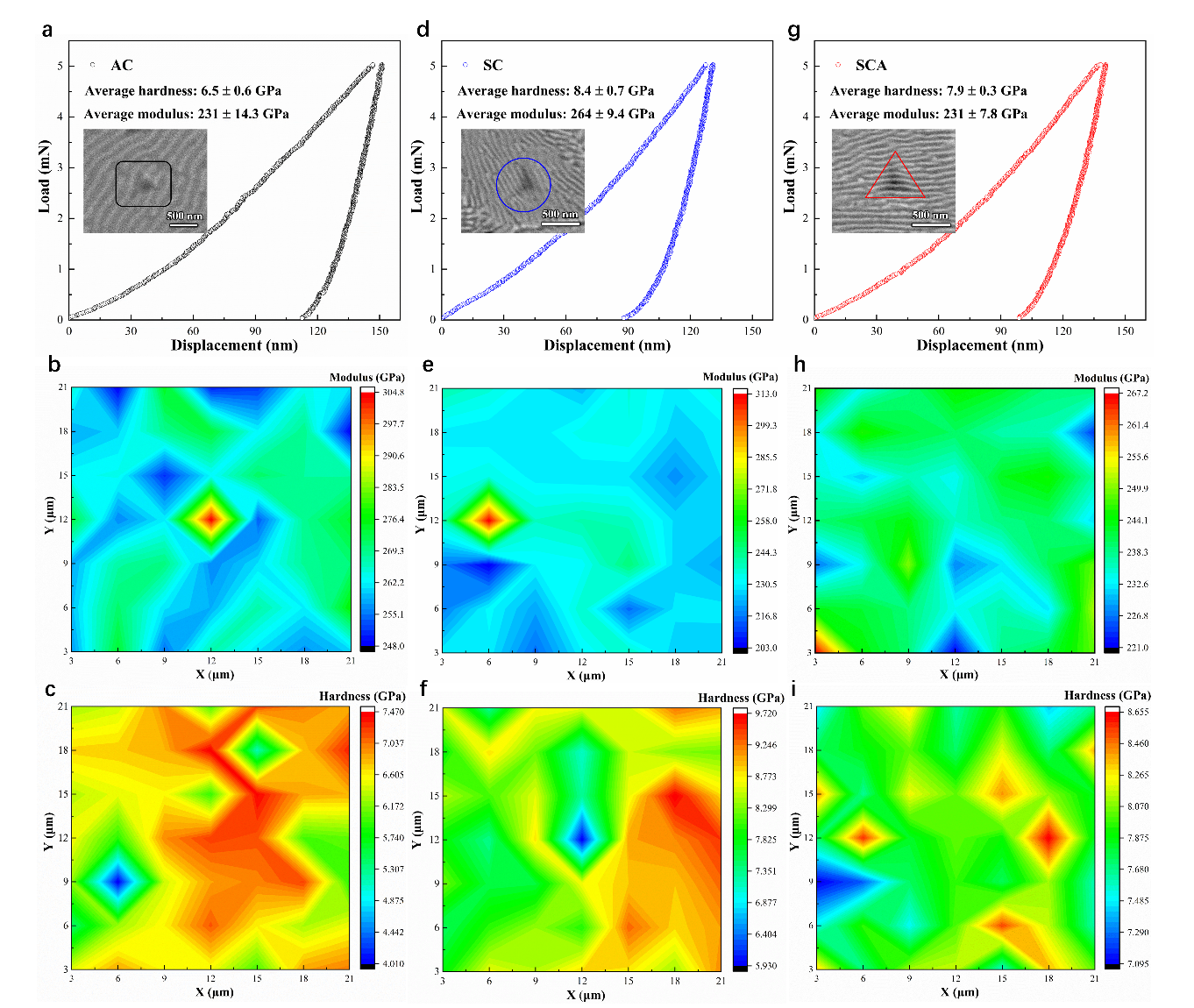


**Figure S12 Comparisons of the hardness and reduced modulus (*E_r_*) between AC, SC and SCA alloys from nanoindentation.** The Load-Displacement curves of **a** AC, **b** SC, and **c** SCA alloy. Insets exhibit the locations of the indentation. Contour-line maps of modulus and hardness for **b-c** AC, **e-f** SC, and **h-i** SCA alloy.

**Supplementary Text 4 | Deformation mechanisms of the AC and SC alloys**

For the AC alloy (**Figure S13, a_1_-a_3_**), only dense dislocations, planar-slip bands, and dislocation pile-ups are characterized in the FCC lamellae during deformation. As reported in previous studies, dislocations are primarily generated and stored within the grain interior in the alloys with coarse grains, exhibiting a typical dislocation slip-dominated deformation mechanism.^[70]^ In this context, the coarse-eutectic layers can be analogized to coarse grains. They facilitate the efficient accumulation of dislocations within the lamellae interiors, giving rise to a planar dislocation-dominated deformation mode, which is responsible for the subsequent substantial ductility. At the strains of 3% and 9% in the SC alloy, the sliding dislocations, extended SFs, and planar faults on the primary {111} plane are activated to accommodate plastic deformation (**Figure S13, b_1_-b_3,_ and S14**). Since thinner lamellae have higher flow stress, this trend drives the leading and trailing partials of a full dislocation further apart, facilitating dislocation splitting at interfaces more readily in thinner lamellae. These thinner lamellae could act as sources of partial dislocations and SFs.^[5]^ Upon further deformation, these SFs are known to serve as a prerequisite for twin nucleation,^[71]^ which typically unites into twins predominantly on the primary slip system. Consequently, it can be concluded that the predominant defect structures, such as dislocations and SFs, govern the deformation behavior of the SC alloy.

As shown in Figure 1G, the compressive engineering stress-strain curves of the AC and SC alloys hardly show strong differences in fracture strains but exhibit significant variations in their yielding behavior. The AC EHEA exhibits a yield strength of 1.3 GPa. In contrast, the ultra-fine grain SC alloy, with lamellae as thick as those in 3D printing,^[5]^ exhibits a modest rise in yield strength up to 2.1 GPa, a result of the Hall-Petch strengthening mechanisms.^[72]^


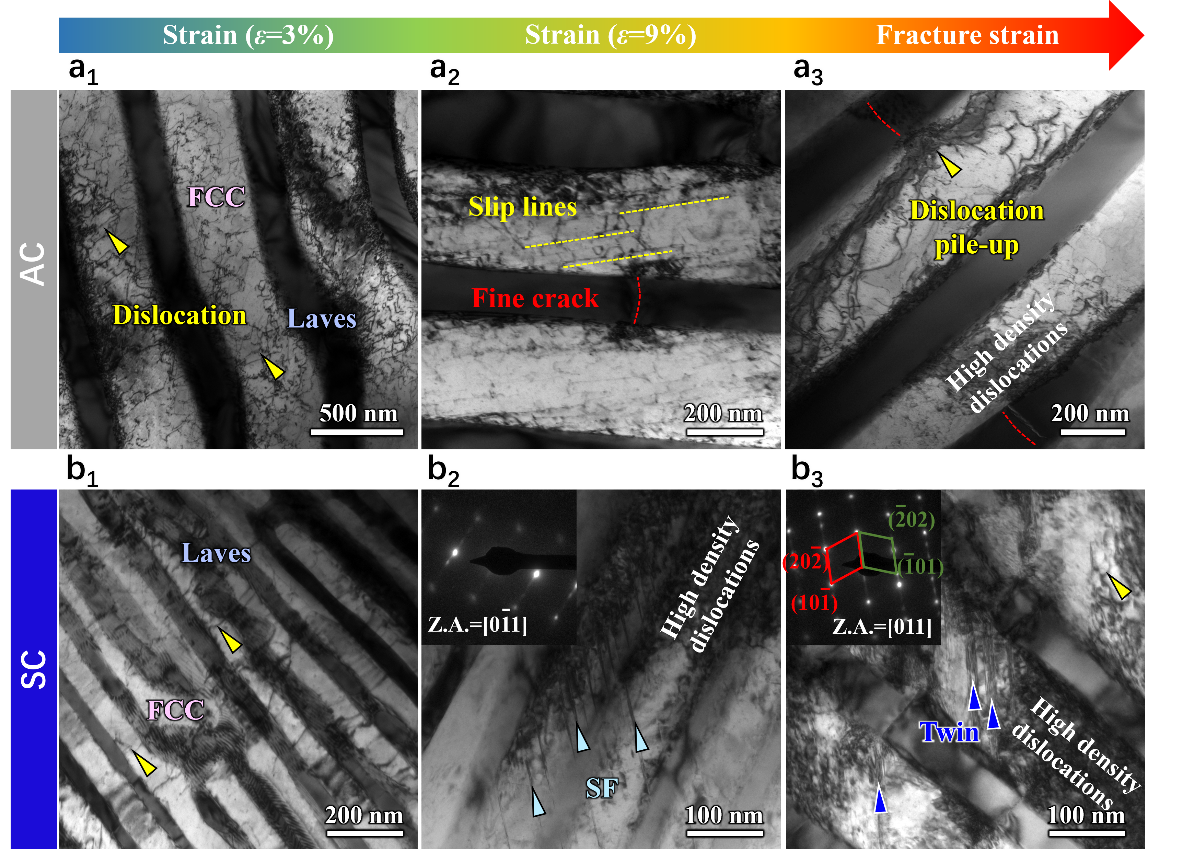


**Figure S13 Deformation mechanisms of AC and SC alloys.** Deformation microstructure of the AC alloy at the **a_1_** 3%, **a_2_** 9%, and **a_3_** fracture strain reveals the slips and pile-up of dislocations (yellow triangle). In the SC alloy, **b_1_** at the 3% strain, rapid slip of dislocations from the FCC matrix to phase interface and planar faults pinning in the FCC matrix. **b_2_** Parallel SFs (light blue triangle, Figure S13b_1,_ and S14) are activated as strained of 9% accompanied by high-density dislocations in the soft FCC lamellae. The inset shows the corresponding streak SAED patterns in illustration and high-density dislocations in the soft FCC lamellae. **b_3_** At the fracture strain, activations of twins (dark blue triangle) along with corresponding twin SAED patterns in illustration, and high-density dislocations in the soft FCC lamellae and phase interfaces.


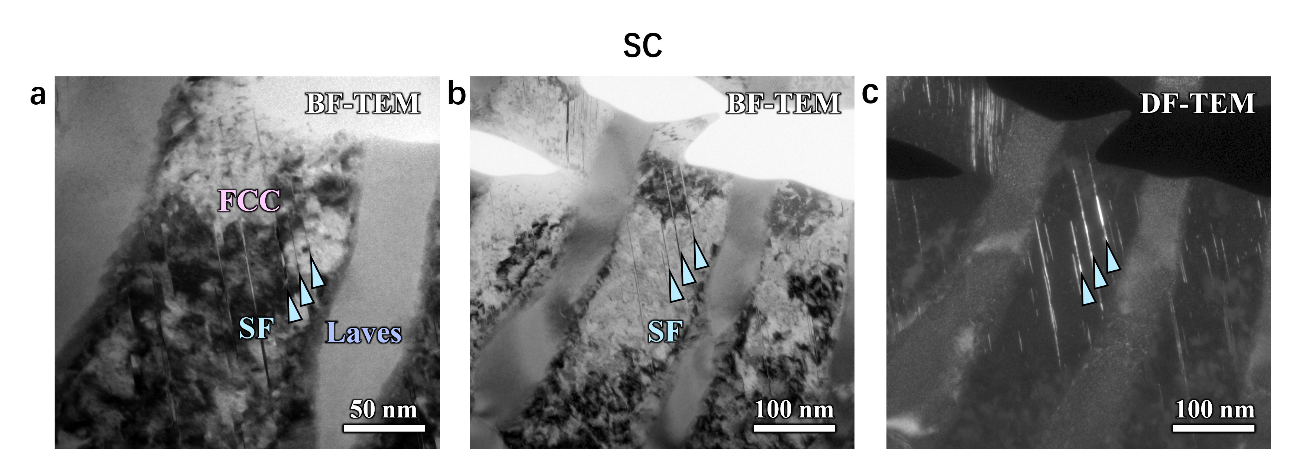


**Figure S14** Microstructure of **a-c** deformation-induced SFs at the 9% strains in the SC alloy.

**Table S1** Compositions of FCC, Laves, and D0_22_ phases in three alloys

| Sample condition | phase | Element (at%) | | | | |
| --- | --- | --- | --- | --- | --- | --- |
|  |  | Co | Cr | Fe | Ni | Ta |
| Nominal | Nominal | 22.7 | 22.7 | 22.7 | 22.7 | 9.2 |
| AC | FCC | 22.10±0.81 | 25.56±1.30 | 25.86±1.20 | 24.46±1.11 | 3.01±0.45 |
|  | Laves | 24.30±0.93 | 18.11±1.46 | 20.10±0.58 | 18.25±0.91 | 19.25±0.50 |
| SC | FCC | 22.03±1.04 | 25.36±2.13 | 26.93±0.88 | 23.34±0.72 | 2.34±0.53 |
|  | Laves | 24.23±0.63 | 16.97±2.89 | 18.94±0.71 | 17.26±0.58 | 22.59±2.56 |
| SCA | FCC | 22.23±1.60 | 25.08±0.58 | 27.04±1.05 | 23.52±1.43 | 2.13±0.41 |
|  | L1_2_ | 20.80±1.10 | 11.50±0.80 | 12.00±0.80 | 38.80±1.30 | 16.90±1.00 |
|  | Laves | 24.14±1.77 | 15.80±0.92 | 19.03±0.88 | 18.77±1.23 | 22.26±1.33 |
|  | D0_22_ | 15.12±0.65 | 12.28±0.79 | 11.79±0.97 | 47.78±1.88 | 13.03±0.58 |

^1)^ The FCC and Laves lamellae from SC and SCA alloys, and L1_2_ precipitated compositions are extracted from APT results (Figure S4 and S6). The compositions of FCC and Laves lamellae from the AC alloy and D0_22_ precipitations in the SCA alloy are extracted from EDS maps (Figure S5 and S8). Errors represent the standard deviation.

**Table S2** Summary of data in Figure 1f, including the yield strength (*σ*_y_), plastic strain (*ε*_p_), ultimate stress (*σ*_US_), and the product of plasticity and ultimate strength (*ε*_p_ × *σ*_US_) for various dual-phase materials.

| Materials | | *σ*_y_ (GPa) | *σ*_US_  (GPa) | *ε*_p_  (%) | *σ*_US_×*ε*_p_  (GPa) | Refs. |
| --- | --- | --- | --- | --- | --- | --- |
| BCC+Laves | Nb_30_Ti_40_Zr_30_ | 0.675 | - | >50 | - | ^[73]^ |
|  | Cr_20_Nb_30_Ti_40_Zr_10_ | 1.005 | 1.435 | 10.9 | 0.15 |  |
|  | Al_15_Cr_20_Nb_15_Ti_40_Zr_10_ | 1.750 | 1.820 | 3.8 | 0.07 |  |
|  | Al_23_Cr_20_Nb_15_Ti_32_Zr_10_ | - | 1.530 | 0 | - |  |
|  | Al_28_Cr_20_Nb_15_Ti_27_Zr_10_ | - | 1.515 | 0 | - |  |
|  | Al_33_Cr_20_Nb_15_Ti_22_Zr_10_ | - | 0.685 | 0 | - |  |
|  | CrNbTiZrAl_0.25_ | - | 1.280 | 0 | - | ^[74]^ |
|  | CrNbTiZrAl_0.5_ | - | 0.600 | 0 | - |  |
|  | CrNbTiZrAl_0.75_ | - | 0.410 | 0 | - |  |
|  | CrNbTiZrAl_1_ | - | 0.620 | 0 | - |  |
|  | CrNbTiZrAl_1.25_ | - | 0.430 | 0 | - |  |
| FCC+Laves | CoFeNi_2_V_0.5_ | 0.172 | - | >80 | - | ^[75]^ |
|  | CoFeNi_2_V_0.5_Nb_0.2_ | 0.660 | - | >80 | - |  |
|  | CoFeNi_2_V_0.5_Nb_0.4_ | 0.776 | 2.267 | 32 | 0.72 |  |
|  | CoFeNi_2_V_0.5_Nb_0.6_ | 1.806 | 2.101 | 7.8 | 0.16 |  |
|  | CoFeNi_2_V_0.5_Nb_0.75_ | 2.073 | 2.232 | 3.4 | 0.07 |  |
|  | CoFeNi_2_V_0.5_Nb_1_ | 2.108 | 2.280 | 2.5 | 0.08 |  |
|  | CoFeNi_2_V_0.5_Nb_0.75_ | 2.073 | 2.232 | 3.4 | 0.07 | ^[76]^ |
|  | Fe_55_Cr_15_Ni_30_ | 0.200 | - | >70 | - | ^[24]^ |
|  | Fe_55_Cr_15_Ni_27_Nb_3_ | 0.400 | - | >70 | - |  |
|  | Fe_55_Cr_15_Ni_24_Nb_6_ | 0.500 | 0.400 | 32 | 0.13 |  |
|  | Fe_55_Cr_15_Ni_21_Nb_9_ | 0.710 | 0.720 | 30 | 0.22 |  |
|  | Fe_55_Cr_15_Ni_18_Nb_12_ | 0.800 | 0.900 | 10 | 0.09 |  |
|  | Fe_55_Cr_15_Ni_15_Nb_15_ | 1.070 | 1.150 | 8 | 0.09 |  |
| FCC+μ | Co_2_Mo_0.5_Ni_2_VW_0.5_ | 0.925 | - | >50 | - | ^[77]^ |
|  | Co_2_Mo_0.6_Ni_2_VW_0.6_ | 1.411 | 2.108 | 11.8 | 0.25 |  |
|  | Co_2_Mo_0.8_Ni_2_VW_0.8_ | 1.431 | 2.364 | 14.4 | 0.34 |  |
|  | Co_2_Mo_1.0_Ni_2_VW_1.0_ | 1.371 | 2.208 | 16 | 0.35 |  |
|  | Co_2_Mo_1.5_Ni_2_VW_1.5_ | 1.320 | 2.133 | 13.9 | 0.29 |  |
|  | Co_2_Mo_1.75_Ni_2_VW_1.75_ | 1.607 | 2.313 | 9.4 | 0.21 |  |
| BCC+Carbide | MoNbRe_0.5_TaW(TiC)_0.2_ | 1.238 | 1.510 | 2 | 0.03 | ^[78]^ |
|  | MoNbRe_0.5_TaW(TiC)_0.5_ | 1.408 | 1.734 | 5.5 | 0.09 |  |
|  | MoNbRe_0.5_TaW(TiC)_0.8_ | 1.438 | 1.903 | 5.3 | 0.10 |  |
|  | MoNbRe_0.5_TaW(TiC)_1.0_ | 1.496 | 1.943 | 5.1 | 0.10 |  |
|  | MoNbRe_0.5_TaW(TiC)_1.5_ | 1.543 | 1.680 | 1 | 0.02 |  |
|  | Re_0.5_MoNbW(TiC)_0.8_ | 1.000 | 2.400 | 8 | 0.192 | ^[23]^ |
|  | Re_0.5_MoNbW(TiC)_0.9_ | 1.200 | 2.520 | 8 | 0.20 |  |
|  | Re_0.5_MoNbW(TiC)_1.0_ | 1.200 | 2.480 | 6 | 0.15 |  |
|  | Re_0.5_MoNbW(TiC)_0.8_ | 1.340 | 2.347 | 8.9 | 0.21 | ^[79]^ |
| BCC+L2_1_ | Cu_40_Al_20_Ti_20_ (as cast) | 0.860 | 0.980 | 2.5 | 0.02 | ^[80]^ |
|  | Cu_40_Al_20_Ti_20_ (homogenized) | 1.300 | 1.800 | 6.3 | 0.11 |  |
|  | AlCr_1.3_TiNi_2_ | 1.500 | 2.700 | 18 | 0.48 | ^[81]^ |
| BCC+B2 | NiAl-Mo_9.0_Cr_9.0_V_9.0_Fe_9.0_ | 1.832 | 3.183 | 18.1 | 0.57 | ^[82]^ |
|  | NiAl-Mo_9.5_Cr_9.5_V_9.5_Fe_9.5_ | 1.908 | 2.950 | 15.0 | 0.44 |  |
|  | NiAl-Mo_10.0_Cr_10.0_V_10.0_Fe_10.0_ | 1.948 | 3.048 | 17.2 | 0.52 |  |
|  | NiAl-Mo_10.5_Cr_10.5_V_10.5_Fe_10.5_ | 2.151 | 3.313 | 19.1 | 0.63 |  |
|  | NiAl-Mo_11.0_Cr_11.0_V_11.0_Fe_11.0_ | 2.190 | 3.542 | 19.4 | 0.69 |  |
|  | NiAl–Mo_8.7_Cr_8.7_V_8.7_ | 1.300 | 1.700 | 7.6 | 0.13 | ^[83]^ |
|  | NiAl–Mo_14.5_Cr_14.5_V_14.5_ | 1.700 | 2.950 | 12 | 0.35 |  |
|  | NiAl–Mo_10_Cr_10_V_10_Fe_10_ | 1.780 | 3.000 | 10 | 0.30 |  |
|  | Al_1.75_CrFeNi (as cast) | 1.325 | 1.619 | 11.8 | 0.19 | ^[84]^ |
|  | Al_1.75_CrFeNi (aged) | 1.050 | 1.716 | 17.6 | 0.30 |  |
|  | Al_1.0_CrFeNi | 1.244 | 1.716 | 17.6 | 0.30 | ^[85]^ |
|  | Al_1.25_CrFeNi | 1.194 | 1.285 | 8.6 | 0.11 |  |
|  | Al_1.5_CrFeNi | 1.148 | 1.309 | 9.1 | 0.12 |  |
|  | Al_1.75_CrFeNi | 1.276 | 1.911 | 18.9 | 0.36 |  |
| BCC+*σ* | AlCrFeNi | 1.167 | 2.077 | 30.2 | 0.63 | ^[86]^ |
|  | AlCrFeNiTi_0.2_ | 1.556 | 1.977 | 18.4 | 0.36 |  |
|  | AlCrFeNiTi_0.5_ | 1.488 | 1.641 | 11.3 | 0.18 |  |
|  | AlCrFeNiTi | 1.416 | 1.440 | 8.2 | 0.12 |  |
| BCC+Silicide | HfMo_0.5_NbTiV_0.5_ | 1.260 | - | >35 | - | ^[87]^ |
|  | HfMo_0.5_NbTiV_0.5_Si_0.3_ | 1.617 | 2.016 | 18.5 | 0.37 |  |
|  | HfMo_0.5_NbTiV_0.5_Si_0.5_ | 1.787 | 2.052 | 11.9 | 0.24 |  |
|  | HfMo_0.5_NbTiV_0.5_Si_0.7_ | 2.134 | 2.242 | 9.2 | 0.21 |  |
|  | (VNbTiTa)_100_Si_0_ | 0.704 | - | >50 | - | ^[88]^ |
|  | (VNbTiTa)_99.5_Si_0.5_ | 1.023 | 1.800 | 28 | 0.50 |  |
|  | (VNbTiTa)_95_Si_5_ | 1.165 | 1.750 | 14 | 0.24 |  |
|  | (VNbTiTa)_90_Si_10_ | 1.671 | 2.100 | 7 | 0.14 |  |
|  | Mo_0.5_NbHf_0.5_ZrTi | 1.180 | 1.500 | 25 | 0.37 | ^[89]^ |
|  | Mo_0.5_NbHf_0.5_ZrTiSi_0.1_ | 1.400 | 2.100 | 28 | 0.60 |  |
|  | Mo_0.5_NbHf_0.5_ZrTiSi_0.3_ | 1.420 | 2.060 | 23 | 0.47 |  |
|  | Mo_0.5_NbHf_0.5_ZrTiSi_0.5_ | 1.680 | 2.000 | 22.5 | 0.45 |  |
|  | Mo_0.5_NbHf_0.5_ZrTiSi_0.7_ | 1.660 | 1.980 | 12 | 0.24 |  |
|  | Mo_0.5_NbHf_0.5_ZrTiSi_0.9_ | 1.700 | 2.070 | 9 | 0.19 |  |
| BMGs | Ti_40_Zr_25_Ni_3_Cu_12_Be_20_ (Ф1) ^1)^ | 1.440 | 1.651 | 13.5 | 0.22 | ^[90]^ |
|  | Ti_40_Zr_25_Ni_3_Cu_12_Be_20_ (Ф3) | 1.583 | 1.710 | 6.9 | 0.12 |  |
|  | Ti_40_Zr_25_Ni_3_Cu_12_Be_20_ (Ф6) | 1.753 | 1.858 | 1.2 | 0.02 |  |
|  | Zr_46_Cu_46_Al_8_ | 1.673 | 1.931 | 3.6 | 0.07 | ^[91]^ |
|  | Zr_46_(Cu_4.5_/_5.5_Ag_1/5.5_)_46_A_l8_ | 1.822 | 2.163 | 6.0 | 0.13 |  |
|  | Zr_45_(Cu_4.5/5.5_Ag_1/5.5_)_48_Al_7_ | 1.839 | 2.105 | 28.1 | 0.59 |  |
| RHEAs | MoNbTaTi | 1.150 | - | >30 | - | ^[92]^ |
|  | WNbTaTi | 0.900 | - | >30 | - |  |
|  | NbTaTi | 0.450 | - | >30 | - |  |
|  | CrMoTaTi | 1.600 | 2.200 | 7.5 | 0.16 |  |
|  | CrMoNbTi | 1.450 | 1.750 | 5 | 0.09 |  |
|  | HfNbTaTi | 0.700 | - | >30 | - |  |
|  | AlNbTaTi | 0.900 | - | >30 | - |  |
|  | HfMoTaTiZr | 1.600 | 1.750 | 4 | 0.07 | ^[93]^ |
|  | HfMoNbTaTiZr | 1.512 | 1.810 | 12 | 0.25 |  |
|  | TiNbMoTaW | 1.343 | 2.005 | 14.1 | 0.28 | ^[94]^ |
|  | TiVNbMoTaW | 1.515 | 2.135 | 10.6 | 0.22 |  |
|  | NbMoTaW | 1.058 | 1.211 | 1.5 | 0.02 | ^[95]^ |
|  | VNbMoTaW | 1.246 | 1.270 | 0.5 | 0.01 |  |
| FCC+Laves | AC | 1.310 | 2.190 | 12 | 0.26 | This work |
|  | SC | 2.090 | 2.630 | 13.4 | 0.35 |  |
|  | SCA | 2.600 | 3.200 | 13.6 | 0.44 |  |

^1)^ *Ф*1: the diameter is 1 mm; *Ф*3: the diameter is 3 mm; *Ф*6: the diameter is 6 mm.

**Table S3** Mixing enthalpy for CoCrFeNiTa_0.4_ eutectic alloy

| Δ*H*^mix^/kJ∙mol^-1^ | | | | | | | | | |
| --- | --- | --- | --- | --- | --- | --- | --- | --- | --- |
| Ta-Co | Ta-Cr | Ta-Fe | Ta-Ni | Ni-Co | Ni-Cr | Ni-Fe | Co-Fe | Co-Cr | Cr-Fe |
| -24 | -7 | -15 | -29 | 0 | -7 | -2 | -1 | -4 | -1 |

**References**

1. D.A. Porter, K. E. Easterling, M.Y. Sherif, “Phase transformations in metals and alloys,” *Boca Raton* (2021): 578

<https://doi.org/10.1201/9781003011804>

1. H. Jiang, K. Han, X. Gao, et al., “A new strategy to design eutectic high-entropy alloys using simple mixture method,” *Mater. Des.* 142, (2018): 101-105.

https://doi.org/10.1016/j.matdes.2018.01.025

1. L. Fan, T. Yang, Y. Zhao, et al., “Ultrahigh strength and ductility in newly developed materials with coherent nanolamellar architectures,” *Nat. Commun.* 11, (2020): 6240.

https://doi.org/10.1038/s41467-020-20109-z

1. M.S. Titus, A. Mottura, G. Babu Viswanathan, et al., “High resolution energy dispersive spectroscopy mapping of planar defects in L1_2_-containing Co-base superalloys,” *Acta Mater.* 89, (2015): 423-437.

<https://doi.org/10.1016/j.actamat.2015.01.050>

1. A. Suzuki, “High-temperature strength and deformation of γ/γ′ two-phase Co–Al–W-base alloys,” *Acta Mater.* 56, (2008): 1288-1297.

<https://doi.org/10.1016/j.actamat.2007.11.014>

1. Y.M. Eggeler, J. Müller, M.S. Titus, et al., “Planar defect formation in the γ′ phase during high temperature creep in single crystal CoNi-base superalloys,” *Acta Mater.* 113, (2016): 335-349.

<https://doi.org/10.1016/j.actamat.2016.03.077>

1. S.K. Makineni, A. Kumar, M. Lenz, et al., “On the diffusive phase transformation mechanism assisted by extended dislocations during creep of a single crystal CoNi-based superalloy,” *Acta Mater.* 155, (2018): 362-371.

<https://doi.org/10.1016/j.actamat.2018.05.074>

1. Y.L. Zhao, T. Yang, Y.R. Li, et al., “Superior high-temperature properties and deformation-induced planar faults in a novel L1_2_-strengthened high-entropy alloy,” *Acta Mater.* 188, (2020): 517-527.

<https://doi.org/10.1016/j.actamat.2020.02.028>

1. B. Décamps, A.J. Morton, M. Condat, “On the mechanism of shear of γ′ precipitates by single (a/2)<110> dissociated matrix dislocations in Ni-based superalloys,” *Philos. Mag. A* 64, (1991): 641-668.

<https://doi.org/10.1080/01418619108204866>

1. I.A. Ovid'ko, R.Z. Valiev, Y.T. Zhu, “Review on superior strength and enhanced ductility of metallic nanomaterials,” *Prog. Mater. Sci.* 94, (2018): 462-540.

<https://doi.org/10.1016/j.pmatsci.2018.02.002>

1. H. Idrissi, K. Renard, L. Ryelandt, et al., “On the mechanism of twin formation in Fe–Mn–C TWIP steels,” *Acta Mater.* 58, (2010): 2464-2476.

<https://doi.org/10.1016/j.actamat.2009.12.032>

1. A. Misra, J.P. Hirth, R.G. Hoagland, “Length-scale-dependent deformation mechanisms in incoherent metallic multilayered composites,” *Acta Mate.* 53, (2005): 4817-4824.

<https://doi.org/10.1016/j.actamat.2005.06.025>

1. N. Yurchenko, E. Panina, S. Zherebtsov, et al., “Design and characterization of eutectic refractory high entropy alloys,” *Mater.* 16, (2021): 101057.

<https://doi.org/10.1016/j.mtla.2021.101057>

1. M. Zhu, L. Yao, Y. Liu, et al., “Microstructure evolution and mechanical properties of a novel CrNbTiZrAl_x_ (0.25≤x≤1.25) eutectic refractory high-entropy alloy,” *Mater. Lett.* 272, (2020): 127869.

<https://doi.org/10.1016/j.matlet.2020.127869>

1. L. Jiang, Y. Lu, Y. Dong, et al., “Effects of Nb addition on structural evolution and properties of the CoFeNi_2_V_0.5_ high-entropy alloy,” *Appl. Phys. A* 119, (2015): 291-297.

<https://doi.org/10.1007/s00339-014-8964-4>

1. L. Jiang, Y. Lu, W. Wu, et al., “Microstructure and mechanical properties of a CoFeNi_2_V_0.5_Nb_0.75_ eutectic high entropy alloy in as-cast and heat-treated conditions,” *J. Mater. Sci. Technol.* 32, (2016): 245-250.

<https://doi.org/10.1016/j.jmst.2015.08.006>

1. H. Jiang, H. Zhang, T. Huang, et al., “Microstructures and mechanical properties of Co_2_MoxNi_2_VW_x_ eutectic high entropy alloys,” *Mater. Des.* 109, (2016): 539-546.

<https://doi.org/10.1016/j.matdes.2016.07.113>

1. Q. Wei, G. Luo, J. Zhang, et al., “Designing high entropy alloy-ceramic eutectic composites of MoNbRe_0.5_TaW(TiC)_x_ with high compressive strength,” *J. Alloys Compd.* 818, (2020): 152846.

<https://doi.org/10.1016/j.jallcom.2019.152846>

1. Q.Q. Wei, X.D. Xu, G.M. Li, et al., “A carbide-reinforced Re_0.5_MoNbW(TaC)_0.8_ eutectic high-entropy composite with outstanding compressive properties,” *Scr. Mater.* 200, (2021): 113909.

<https://doi.org/10.1016/j.scriptamat.2021.113909>

1. L. Wang, J. Wang, H. Niu, et al., “BCC+L2_1_ dual-phase Cu_40_Al_20_Ti_20_V_20_ near-eutectic high-entropy alloy with a combination of strength and plasticity,” *J. Alloys Compd.* 908, (2022): 164683.

<https://doi.org/10.1016/j.jallcom.2022.164683>

1. M. Wang, Y. Lu, T. Wang, et al., “A novel bulk eutectic high-entropy alloy with outstanding as-cast specific yield strengths at elevated temperatures,” *Scr. Mater.* 204, (2021): 114132.

<https://doi.org/10.1016/j.scriptamat.2021.114132>

1. L. Wang, Y. Su, C. Yao, et al., “Microstructure and mechanical property of novel NiAl-based hypoeutectic/eutectic/hypereutectic high-entropy alloy,” *Intermetallics* 143, (2022): 107476.

<https://doi.org/10.1016/j.intermet.2022.107476>

1. L. Wang, C. Yao, J. Shen, et al., “A new method to design eutectic high-entropy alloys by determining the formation of single-phase solid solution and calculating solidification paths,” *Mater. Sci. Eng. A* 830, (2022): 142325.

<https://doi.org/10.1016/j.msea.2021.142325>

1. M. Wang, Z. Wen, J. Liu, et al., “Novel ultra-high aluminum content eutectic high entropy alloys with duplex-reinforced phases and labyrinthine structure,” *J. Mater. Sci.* 57, (2022): 11365-11375.

<https://doi.org/10.1007/s10853-022-07322-5>

1. M. Wang, Z. Wen, J. Liu, et al., “Labyrinthine structure Al_x_CrFeNi (x≥1) eutectic high entropy alloys with duplex reinforced phases,” *J. Alloys Compd.* 918, (2022): 165441.

<https://doi.org/10.1007/s10853-022-07322-5>

1. L.S. Y., F.D. Chen, X.B. Tang, et al., “Effect of Ti on the microstructure and mechanical properties of AlCrFeNiTi_x_ eutectic high-entropy alloys,” *J. Mater. Eng. Perform.* 31, (2022): 8294-8303.

<https://doi.org/10.1007/s11665-022-06825-1>

1. Y. Liu, Y. Zhang, H. Zhang, et al., “Microstructure and mechanical properties of refractory HfMo_0.5_NbTiV_0.5_Si_x_ high-entropy composites,” *J. Alloys Compd.* 694, (2017): 869-876.

<https://doi.org/10.1016/j.jallcom.2016.10.014>

1. Z.Q. Xu, Z.L. Ma, Y. Tan, et al., “Effects of Si additions on microstructures and mechanical properties of VNbTiTaSi refractory high-entropy alloys,” *J. Alloys Compd.* 900, (2022): 163517.

<https://doi.org/10.1016/j.jallcom.2021.163517>

1. N.N. Guo, L. Wang, L.S. Luo, et al., “Microstructure and mechanical properties of refractory high entropy (Mo_0.5_NbHf_0.5_ZrTi)_BCC_/M_5_Si_3_ in-situ compound,” *J. Alloys Compd.* 660, (2016): 197-203.

<https://doi.org/10.1016/j.jallcom.2015.11.091>

1. Y.J. Huang, J. Shen, J.F. Sun, “Bulk metallic glasses: Smaller is softer,” *Appl. Phys. Lett.* 90, (2007): 081919.

<https://doi.org/10.1063/1.2696502>

1. Q.K. Jiang, X.D. Wang, X.P. Nie, et al., “Zr-(Cu, Ag)-Al bulk metallic glasses,” *Acta Mater.* 56, (2008): 1785-1796.

<https://doi.org/10.1016/j.actamat.2007.12.030>

1. F.G. Coury, M. Kaufman, A.J. Clarke, “Solid-solution strengthening in refractory high entropy alloys,” *Acta Mater.* 175, (2019): 66-81.

<https://doi.org/10.1016/j.actamat.2019.06.006>

1. C.-C. Juan, M.-H. Tsai, C.-W. Tsai, et al., “Enhanced mechanical properties of HfMoTaTiZr and HfMoNbTaTiZr refractory high-entropy alloys,” *Intermetallics* 62, (2015): 76-83.

<https://doi.org/10.1016/j.intermet.2015.03.013>

1. Z.D. Han, N. Chen, S.F. Zhao, et al., “Effect of Ti additions on mechanical properties of NbMoTaW and VNbMoTaW refractory high entropy alloys,” *Intermetallics* 84, (2017): 153-157.

<https://doi.org/10.1016/j.intermet.2017.01.007>

1. O.N. Senkov, G.B. Wilks, J.M. Scott, et al., “Mechanical properties of Nb_25_Mo_25_Ta_25_W_25_ and V_20_Nb_20_Mo_20_Ta_20_W_20_ refractory high entropy alloys,” *Intermetallics* 19, (2011): 698-706.

<https://doi.org/10.1016/j.intermet.2011.01.004>
